# Comprehensive Speciation and Computational Study of Cu^2+^ and Zn^2+^ Complexation with O-Phosphorylethanolamine and O-Phosphorylcholine in Aqueous Solution

**DOI:** 10.3390/molecules30193923

**Published:** 2025-09-29

**Authors:** Federica Carnamucio, Chiara Abate, Massimiliano Cordaro, Claudia Foti, Salvatore Donato, Franz Saija, Giuseppe Cassone, Ottavia Giuffrè

**Affiliations:** 1 Department of Pharmaceutics, Center for Pharmaceutical Engineering and Sciences, School of Pharmacy, Virginia Commonwealth University, 410 N 12th St., Richmond, VA 23284, USA; carnamuciof@vcu.edu; 2 Department of Chemical, Biological, Pharmaceutical, and Environmental Sciences, University of Messina, 31 Viale F. Stagno d’Alcontres, 98166 Messina, Italy; cabate@unime.it (C.A.); mcordaro@unime.it (M.C.); cfoti@unime.it (C.F.); salvatore.donato@studenti.unime.it (S.D.); 3 Institute for Chemical-Physical Processes, National Research Council of Italy (CNR-IPCF), 37 Viale F. Stagno d’Alcontres, 98158 Messina, Italy; saija@ipcf.cnr.it

**Keywords:** O-phosphorylethanolamine, O-phosphorylcholine, Cu(II), Zn(II), speciation study, quantum-mechanical calculations

## Abstract

An extensive study on the interactions between O-phosphorylethanolamine (PEA) and O-phosphorylcholine (PPC), Cu^2+^ and Zn^2+^, is thoroughly described. The formation constants were determined at different temperatures (15 ≤ t/°C ≤ 37) and ionic strengths (0.15 ≤ I/mol L^−1^ ≤ 0.97) by potentiometric titrations. For the Zn^2+^-PEA/-PPC systems, speciation models were also confirmed by ^1^H NMR titrations at t = 25 °C and I = 0.15 mol L^−1^ in NaCl. Sequestering abilities were calculated under different temperatures and physiological conditions. Density Functional Theory (DFT) calculations along with enhanced sampling of the conformational space were performed aimed to better elucidate the Cu^2+^-, Zn^2+^- PEA/PPC molecular interactions and their relative stabilities. Overall, both experiments and computer simulations showed that the complex species involved in the Cu^2+^–PEA system exhibited a significant and selective stability, particularly in conditions simulating cerebrospinal fluid. While the binding molecular mechanisms were elucidated via DFT supplemented by automized conformational search, the computational binding energies trend qualitatively follows the experimental log*K* behavior across the Cu^2+^-, Zn^2+^- PEA/PPC complexes. These results highlight the potential physiological role of PEA in modulating free copper levels and regulating its redox activity in pathological conditions, such as Wilson’s Disease (WD).

## 1. Introduction

**Figure 1:**
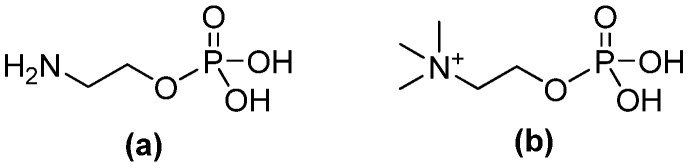
Chemical structure of (**a**) O-phosphorylethanolamine (PEA) and (**b**) O-phosphorylcholine (PPC).

Lipids serve as components of cell membranes and are based on differences in their backbone and polar head groups [1,2]. Specifically, phospholipids are the elementary building block of cell membranes and are involved in several functions, such as in structure, signaling, and trafficking [3]. O-phosphorylethanolamine (PEA, Figure 1a) and O-phosphorylcholine (PPC, Figure 1b) are two zwitterionic constituents of the head groups of biological lipid membranes and represent the most abundant phospholipids in eukaryotic membranes [3,4,5,6]. The latter (PPC), also known as “phosphocholine”, is a small molecule consisting of a negatively charged phosphate bonded to a positively charged choline [7]. PPC is expressed in prokaryotes (on the cell wall of the Gram-positive Streptococcus pneumoniae, or also in *Haemophilus influenza*, and *Neisseria* spp.), eukaryotes (i.e., fungi, protozoa, insects, and nematodes) [7], and it is also one of the major natural phospholipids in the body [8]. PPC has antigenic activity in various disease-causing parasites (i.e., gastrointestinal and filarial nematodes) and protozoa (i.e., *Trypanosoma* and *Leishmania*) and induces an immune response preventing respiratory infections; although it is produced by bacteria and hosts, it interacts with both, and its bioactivity has not yet been fully elucidated [7].

On the other hand, in addition to being a constituent of phospholipids, PEA is a basic building block of sphingomyelin and it is found in the brain at mmol L^−1^ range (~1.4 mmol kg^−1^_ww_) [9]. PEA acts as a precursor to choline-containing compounds, which are important in the formation and maintenance of cell membranes [9]. Both PPC and PEA can act as ligands and give rise to interactions, namely host–guest interactions, which are attracting growing interest in clinical applications [10,11,12,13]. For instance, Cuellar et al. [10] have advantageously used PEA to improve delivery of Amphotericin B, a wide-spectrum antibiotic for the treatment of fungal infections. In addition, PPC and PEA may constitute carriers, that are lipid nanoparticles (LNPs), of systems containing siRNA, a specific type of double-stranded RNA fragment [11].

However, alteration in the level of PEA and/or PPC in the cerebrospinal fluid (CSF), subcortical and cortical cortexes indicate neurodegenerative disorders and intracranial tumors [9]. PEA accumulation plays a very significant role in terms of immunosuppression in the tumor microenvironment [14]. In particular, an increased level of PEA can inhibit choline and acetylcholine synthesis, and an increased level of PPC in CSF was observed in Alzheimer’s patients compared with the normal value of 1.28 μmol L^−1^ [4]. On the other hand, it is well-known that metals of biological interest, such as copper and zinc, are extremely valuable to the organism, where they are involved in different metabolic pathways; their disruption in homeostasis can result in a variety of detrimental effects, pathogenic conditions and, hence, diseases, such as asthma, diabetes, cancer, and Neurodegenerative Diseases (NDs) (i.e., Alzheimer’s Disease (AD), Wilson’s Disease (WD), and Menkes Disease (MD)) [15,16,17,18,19,20,21,22]. Indeed, copper and zinc are involved in the regulation of the physiological and pathological activity of major proteins in AD and/or other NDs [18,19,20,21,22,23]. For instance, Cu^2+^ and Zn^2+^ can catalyze toxic amyloid beta protein (Aβ) in the brains of AD patients, inducing the formation of toxic amyloid fibrils and complexes (i.e., Cu^2+^/Cu^+^- Aβ) and causing neuroinflammation and degeneration [22,24,25]. In particular, copper is an essential redox-active element with non-enzymatic and, thus, structural, signaling, and regulatory properties; it may also be involved in modulating membrane receptor–ligand interactions, control kinase, and phosphatase functions [18,21]. However, due to its redox activity, it could lead to an increase in overall oxidative stress. Specifically, Cu^2+^ or Cu^2+^- Aβ can be reduced to Cu^+^ form that can partially reduce O_2_ to provide Reactive Oxygen Species (ROS), including superoxide (O_2_^−^**•**), hydrogen peroxide (H_2_O_2_), and hydroxyl radical (HO**•**) [21,24].

Therefore, due to its potent redox effects, Cu^2+^ is receiving increasing attention. However, it is crucial to understand whether its toxicity is due to deficiency, accumulation, or both phenomena [18]. Chelation therapy is a strategic approach to reducing Cu^2+^ levels; specifically, it focuses on the selective coordination of ions and the subsequent formation of stable complex species. In this context, a distinction between chelators and ionophores can be made. For instance, in the case of WD, while d-penicillamine or triethyltetramine (or trientine) are Cu^2+^-chelators and, as such, act by sequestering the metal intracellularly, thiosemicarbazones and clioquinol have an antitumor effect through ionophoric functions. However, an increase in intracellular Zn concentration could also be attributed to clioquinol. In light of these deepened analyses [18,21,22,26,27,28], it is worth mentioning the main metal chelators in NDs, such as the first Cu chelator introduced in the treatment of WD, 2,3-dimerkaptopropranol (British anti-Lewisite, BAL), d-penicillamine, and triethyltetramine (or trientine). Nevertheless, when complexed with Cu, they cause increased urinary Cu excretion; furthermore, an increase in the free metal ions in the blood and brain has been attributed to d-penicillamine. Tetrathiomolybdate is an alternative to trientine; however, its limited use is due to the instability of the ammonium formulation, and the combination of bis-choline tetrathiomolybdate and metal complexes, with proteins, warrants further studies, as high doses can result in the precipitation of insoluble Cu-Mo complexes in the liver and lower doses can increase Cu excretion.

Therefore, so far, the metal chelation strategy has not produced the expected therapeutic results, and its use is often controversial. After all, finding novel chelating ionophores, having high affinity for specific metal ions and resulting in non-toxic complexes with negligible side effects, is of great interest. Thus, further investigation is needed into the use of metal complexes in clinical applications, particularly for NDs [18,24].

In light of the above, for the first time to our knowledge, this paper deals with the study of Cu^2+^ and Zn^2+^ interactions with the constituents of biological membranes, which—in turn—are in contact with physiological solutions containing metal cations, proving a higher stability and selectivity of the complex species in the Cu^2+^–PEA system. This study is also of great importance because metal–ligand interactions provide different structures and stability, also affecting cation transport, lipoprotein formation, and various biochemical processes [29]. The use of computational approach gives the possibility to obtain a molecular structure, sequestering modalities and energy information [30].

In particular, in this paper, to evaluate and elucidate the strength of the interactions of Cu^2+^ and Zn^2+^ with PEA and PPC, whose role in NDs is unknown and undetermined, a comprehensive study including experimental techniques (potentiometry and ^1^H-NMR spectroscopy) and computational methods (Density Functional Theory, DFT) was performed. In more detail, the thermodynamic interaction parameters of the Cu^2+^-PEA, Cu^2+^-PPC, Zn^2+^-PEA, and Zn^2+^PPC systems were investigated under different conditions of temperature (15 ≤ t/°C ≤ 37) and ionic strength (0.15 ≤ I/mol L^−1^ ≤ 0.97). The sequestering ability of PEA and PPC toward Cu^2+^ and Zn^2+^ was determined under various values of temperature and physiological conditions, while DFT calculations combined with enhanced sampling of the conformational space were employed to clarify the microscopic binding modes and the relative energetic gains of PEA and PPC with Cu^2+^ and Zn^2+^. This in-depth investigation aims to use the acquired speciation data to provide information on the linkages connecting the macroscopic thermodynamic properties and the molecular behavior under real-world biological conditions, providing clear and relevant evidence and insight into development of metal complexes in clinical trials.

## 2. Results and Discussion

### 2.1. Interaction of PEA and PPC with Cu^2+^

The interaction of Cu^2+^ with PEA and PPC has been investigated via potentiometric titrations across a range of temperatures (15 ≤ t/°C ≤ 37) and ionic strengths (0.15 ≤ I/mol L^−1^ ≤ 0.97). The protonation constants of both ligands, previously investigated [4], along with the hydrolytic constants of Cu^2+^ and the formation constants of the Cu^2+^–Cl^−^ complexes, employed in this study, are reported in Tables S1 and S2 (Supplementary Materials). Speciation models were selected based on multiple criteria, including simplicity, the percentage of species formed, statistical parameters (such as variance, variance ratio, and mean deviation), and their ability to accurately reproduce the experimental data under varying conditions [31]. In these models, the species L corresponds to the fully deprotonated form at both hydroxyl sites. For PEA, L carries two negative charges (L^2−^), with protonation occurring first at the oxygens (LH^−^ and LH_2_^0^) and subsequently at the amine group, giving rise to the positively charged species (LH_3_^+^). For PPC, L similarly represents the deprotonated form at both hydroxyl sites (L^−^), while their protonation leads to the LH^0^ and LH_2_^+^ species.

For both the Cu^2+^–PEA and Cu^2+^–PPC systems, the best-fitting models involve the formation of two predominant complexes, ML and M_2_L(OH)_2_, arising from the following reactions (charges omitted for simplicity):

pM + L + rH ⇌ M_p_LH_r_ (β_M_p_LH_r_) (1)

M + LH_r_ ⇌ MLH_r_ (*K*_MLH_r_) (2)

M_p_(OH)_r_ + L ⇌ M_p_L(OH)_r_ (*K*_M_p_L(OH)_r_) (3)

where r takes values from −4 to +1 and p from 1 to 3. All the formation constants of Cu^2+^-L determined via potentiometry at various temperatures and ionic strengths are summarized in Table 1. A comparison of the formation constant values under different conditions reveals some differences in the complexation behavior of PEA and PPC toward Cu^2+^. In all tested conditions, the Cu^2+^–PEA system forms more stable complexes than Cu^2+^–PPC. For instance, at 25 °C and I = 0.15 mol L^−1^, the log*K* for ML is 5.63 for PEA, vs. just 2.05 for PPC, corresponding to an enhancement in stability by over three orders of magnitude. This difference is even very striking for the di-nuclear hydrolyzed complexes M_2_L(OH)_2_, whose formation constant, at 25 °C and I = 0.15 mol L^−1^, is higher with PEA (log*K* = 6.17) than with PPC (log*K* = 3.89). Temperature influences both systems in a comparable direction, with a slight increase in ML stability for PEA at higher temperatures (from 5.63 to 6.31 between 25 °C and 37 °C), whereas PPC shows smaller variations. Ionic strength has a more pronounced effect on M_2_L(OH)_2_ complexes for both systems: an increase in the ionic strength from 0.15 to 0.97 mol L^−1^ leads to higher log*K* values.

**Figure 2:**
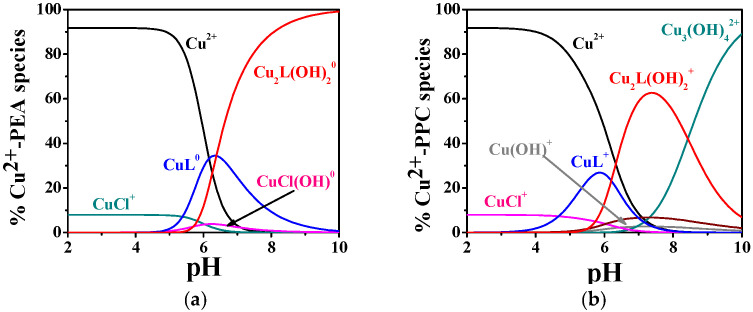
Distribution diagrams of (**a**) Cu^2+^-PEA (L) and (**b**) Cu^2+^-PPC (L) complexes at C_L_ = 4 mmol L^−1^, C_M_ = 2 mmol L^−1^, t = 37 °C, and I = 0.15 mol L^−1^ in NaCl.

By way as an example, some speciation diagrams of Cu^2+^–PEA and Cu^2+^–PPC systems have been reported in Figure 2a,b. The Cu^2+^–PEA system (t = 37 °C, I = 0.15 mol L^−1^) displays the formation of ML^0^ starting from pH 5.5, with a maximum formation percentage of ~35% (Figure 2a). The M_2_L(OH)_2_^0^ complex appears at pH 6.0 and becomes the dominant form around physiological pH (~80% at pH 7.4).

By contrast, Figure 2b, with Cu^2+^–PPC under the same temperature and ionic strength conditions, shows ML^+^ formation beginning at lower pH values (pH 4.0), with a maximum of ~30%. The M_2_L(OH)_2_^+^ complex begins to form at pH 5.5 and reaches ~65% at pH 7.4.

### 2.2. Interaction of PEA and PPC with Zn^2+^

**Figure 3:**
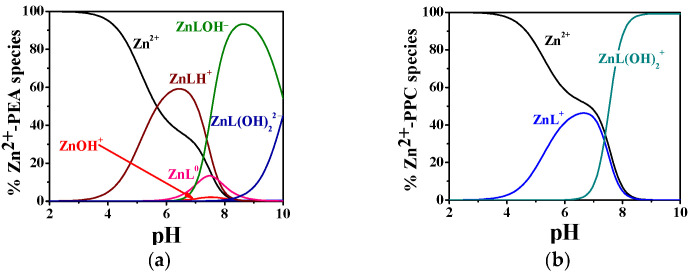
Distribution diagram of (**a**) Zn^2+^-PEA(L) and (**b**) Zn^2+^-PPC(L) complexes at C_L_ = 4 mmol L^−1^, C_M_ = 2 mmol L^−1^, t = 37 °C, and I = 0.15 mol L^−1^ in NaCl.

The complexation behavior of Zn^2+^ with PEA and PPC exhibits considerable differences in both the number of complexes formed and their relative stabilities, as evidenced by the potentiometric data in Table 2 and the distribution diagrams in Figure 3a,b. The hydrolytic constants of Zn^2+^ employed in the model calculations are included in Table S3. The Zn^2+^–PEA system displays a notably more articulated speciation pattern, including ML^0^, protonated (MLH^+^), and two hydrolyzed complexes (MLOH^−^, ML(OH)_2_^2−^), whereas the Zn^2+^–PPC system is characterized by a simpler distribution profile (ML^+^ and ML(OH)_2_^−^). As reported in Table 2, at 25 °C and I = 0.15 mol L^−1^, the log*K* for the formation of ML in the Zn^2+^–PEA system is 4.29, whereas the corresponding value for the Zn^2+^–PPC complex is only 2.41. Although both ligands form hydrolyzed complexes at higher pH, the log*K* values for ML(OH)_2_ are similar (4.55 for PEA and 4.42 for PPC at 25 °C and I = 0.15 mol L^−1^), suggesting comparable stability of the complexes. Nevertheless, the formation of additional complex species in the PEA system, such as MLH^+^ and MLOH^−^, which are not observed in the PPC system, highlights the greater capacity of PEA to stabilize Zn^2+^ through different complexation modes.

Temperature effects were found to be significant, mainly for Zn^2+^-PEA complexes. As an example, going from 25 to 37 °C, the log*K* of the ML^0^ complex in the PEA system increases from 4.29 to 4.90.

As shown in Figure 3a, the MLH^+^ complex dominates the speciation profile between pH 4.0 and 8.0, with a maximum formation percentage exceeding 60% at t = 37 °C. As the pH increases, the speciation shifts toward the formation of ML^0^, followed by a progressive predominance of hydrolyzed complexes, i.e., MLOH^−^ and ML(OH)_2_^2−^, from pH 7.0 onwards. In contrast, the Zn^2+^–PPC system exhibits a much simpler model (Figure 3b), forming only two detectable complexes over the entire pH range investigated. The ML^+^ complex is most prevalent between pH 4.0 and 7.0, with a maximum formation of approximately 45%, while ML(OH)_2_^−^ emerges as the dominant form at pH values above 7.5, becoming the sole complex from pH 8.5 onwards. The restricted speciation and narrower pH-dependent distribution profile point to a reduced complexation ability of PPC toward Zn^2+^.

### 2.3. ^1^H NMR Spectroscopy

**Figure 4:**
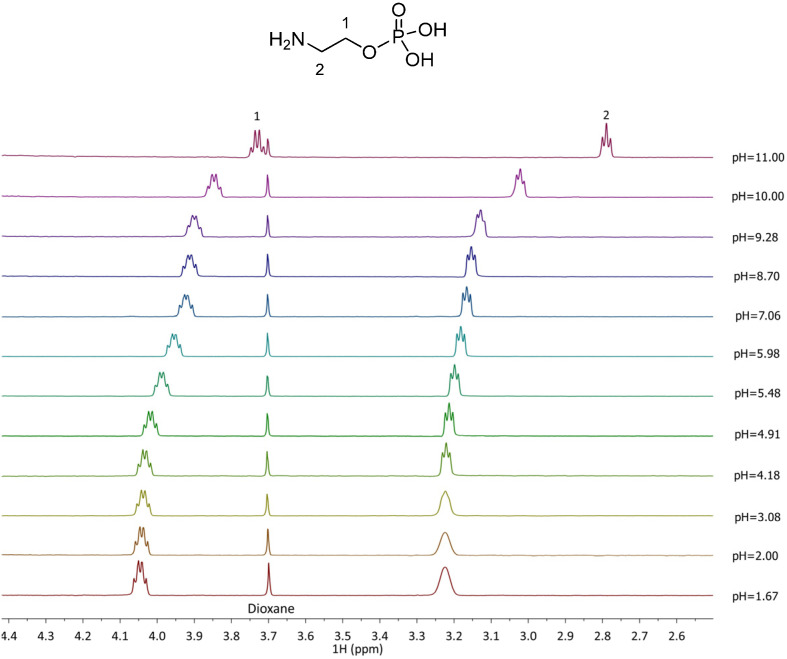
^1^H-NMR spectra of PEA (L) solutions at t = 25 °C, 1.67 ≤ pH ≤ 11.00, C_L_ = 5.5 mmol L^−1^, and I = 0.15 mol L^−1^ in NaCl.

**Figure 5:**
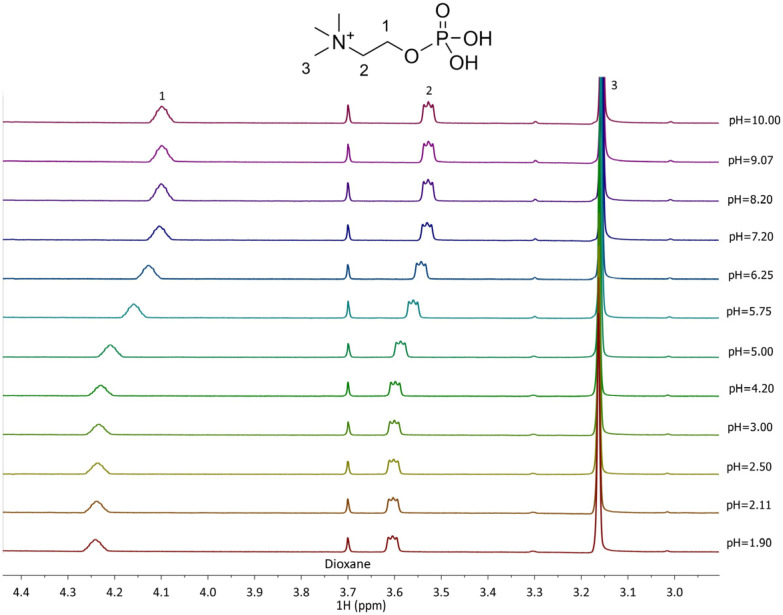
^1^H-NMR spectra of PPC (L) solutions at t = 25 °C, 1.90 ≤ pH ≤ 10.00, C_L_ = 6.4 mmol L^−1^, and I = 0.15 mol L^−1^ in NaCl.

Titrations using ^1^H NMR spectroscopy were conducted for the Zn^2+^–PEA and Zn^2+^–PPC systems with the aim of comparing and confirming the potentiometric results. Due to the paramagnetic properties of Cu^2+^, analogous analyses on the Cu^2+^–L complexes were not feasible. ^1^H NMR spectra of free ligands, PEA and PPC, were first acquired. The proton resonance pattern of free PEA is shown in Figure 4. Two key signals were observed: a triplet of doublets (td) attributed to the CH_2_ protons at position 1, with a chemical shift variation (Δδ) of 0.32 ppm, shifting from 4.05 to 3.73 ppm across the studied pH range; and a triplet (t) corresponding to the CH_2_ protons in position 2, with a Δδ of 0.43 ppm from 3.22 to 2.79 ppm. For PPC (Figure 5), the proton pattern includes a broad multiplet (m) assigned to the CH_2_ in position 1, with a Δδ of 0.14 ppm (4.24–4.10 ppm), and a triplet for CH_2_ in position 2 with a Δδ of 0.07 ppm (3.60–3.53 ppm). Additionally, a singlet at δ = 3.16 ppm, corresponding to the nine ammonium methyl protons, remains essentially unaffected by pH variation.

**Figure 6:**
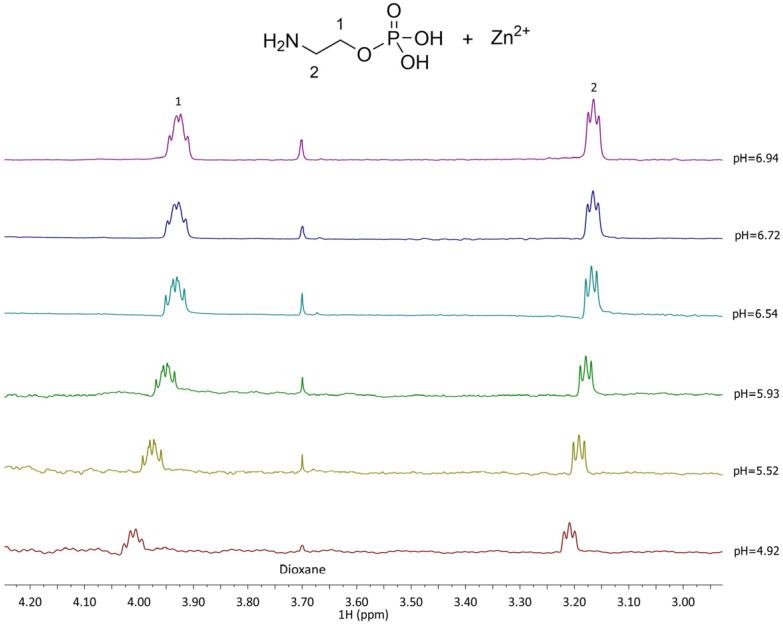
^1^H-NMR spectra of Zn^2+^-PEA (L) at t = 25 °C, 4.92 ≤ pH ≤ 6.94, C_L_ = 5.5 mmol L^−1^, C_M_ = 5.6 mmol L^−1^, and I = 0.15 mol L^−1^ in NaCl.

**Figure 7:**
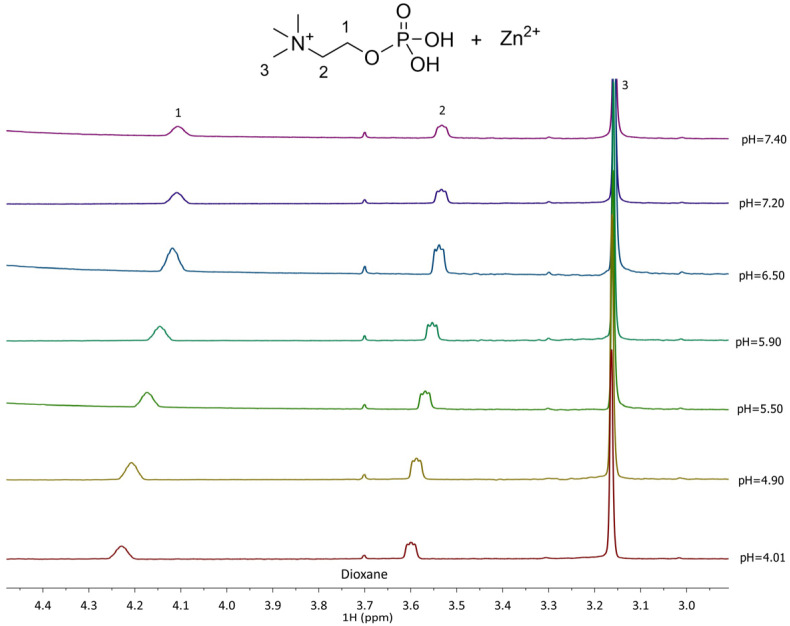
^1^H-NMR of Zn^2+^-PPC (L) at t = 25 °C, 4.01 ≤ pH ≤ 7.40, C_L_ = 6.4 mmol L^−1^, C_M_= 3.6 mmol L^−1^, and I = 0.15 mol L^−1^ in NaCl.

The ^1^H NMR spectra recorded for the Zn^2+^-L complexes show distinct differences from the free L, indicating complex species formation. In the Zn^2+^–PEA system (Figure 6), the CH_2_(1) protons experience deshielding effects depending on the complex formed, with chemical shifts ranging from 3.7 to 4.6 ppm, while CH_2_(2) protons shift from 2.71 to 3.58 ppm. These variations reflect the formation of multiple complexes (MLH^+^, ML^0^, MLOH^−^, and ML(OH)_2_^2−^) in equilibrium, in line with the potentiometric speciation results. In Zn^2+^–PPC system, in contrast, CH_2_(1) and CH_2_(2) shift only slightly, ranging from 3.53 to 3.54 ppm (Figure 7), consistent with the formation of fewer and thus smaller formation percentages of the complex species.

The experimental ^1^H NMR data were processed using HypNMR2008 software to compare and validate the speciation models proposed for both the Zn^2+^–PEA and Zn^2+^–PPC systems. The analysis enabled refinement of the formation constants for the major complexes (with higher formation percentages) detected within the studied pH range, while keeping the stability constants of the minor complexes, previously determined by potentiometric measurements, fixed. Table 3 presents the comparative results from both techniques, revealing a high level of consistency that further validates the reliability of the speciation model.

The agreement between experimental data and model predictions is further illustrated in Figure S1 (Supplementary Materials), which shows the close match between observed and calculated chemical shift vs. pH for both systems. The close correlation between the observed and theoretical data reinforces the robustness of the model. Additionally, the calculated chemical shift values assigned to each individual Zn^2+^ complex species, offering further insight into the coordination environment of the ligands under varying pH conditions, were reported in Table S4.

### 2.4. Dependence of Formation Constant Values on Ionic Strength and Temperature

The formation constants at different ionic strengths were analyzed, as performed for other systems, using the Debye–Hückel-type equation [32]:

(4) $$log\beta ={log\beta }^{0}-0.51z^{*}\;\frac{\sqrt{I}}{1+1.5\sqrt{I}}+CI$$

where β^0^ is the formation constant at infinite dilution, I is the ionic strength, and C is an empirical parameter related to the stoichiometry and charge of the species; z* represents the charge parameter calculated as the sum of the squared charges of reactants minus that of products z* = Σ(charges)^2^_reactants_ − Σ(charges)^2^_products_.

Values of z* are listed in Table 4. The C term is an empirical parameter accounting for short-range ion–ion interactions not captured by the Debye–Hückel approximation. C was obtained by non-linear least-squares fitting of experimental logβ data using the above equation, with a 95% confidence interval.

The formation constants were determined at various ionic strengths ranging from 0.15 to 0.97 mol L^−1^, with the corresponding values of logβ^0^ and C reported in Table 4. These parameters enable the calculation of formation constants across the investigated ionic strength range, facilitating modeling under various conditions. 

The temperature dependence of the formation constants for the Cu^2+^–PEA, Cu^2+^–PPC, Zn^2+^–PEA, and Zn^2+^–PPC complexes was evaluated, as already performed for other systems, using the van’t Hoff equation [33]:

log^*T*^β = logβ_θ_ + ΔH_θ_ (1/θ − 1/T) (5)

where log^*T*^β is the equilibrium constant at temperature T (K), logβ_θ_ is the equilibrium constant at the reference temperature θ = 298.15 K, ΔH_θ_ is the standard enthalpy change, and R is the gas constant. The standard Gibbs free energy change ΔG^0^_θ_ was calculated from logβ_θ_ using ΔG^0^_θ_ = −2.303 R T logβ_θ_ and TΔS^0^_θ_ from TΔS^0^_θ_ = ΔH^0^_θ_ − ΔG^0^_θ_.

The calculated thermodynamic parameters, including enthalpy, entropy, and Gibbs free energy changes at 25 °C, are summarized in Table 4.

The results indicate that for ML formed by Cu^2+^ with both PEA and PPC, complex formation is predominantly driven by entropic contributions, consistent with electrostatic interactions. Conversely, Zn^2+^ complexes exhibit predominantly exothermic enthalpy changes, except for mixed hydrolyzed complex, where the enthalpic term is slightly predominant.

### 2.5. Sequestering Ability

Both Cu^2+^ and Zn^2+^ are essential trace elements involved in numerous physiological processes, yet their dysregulation can lead to cytotoxic effects through mechanisms such as oxidative stress, protein misfolding, and interference with enzymatic activity [34]. In this context, understanding the complexation behavior of biomolecules like PEA and PPC with these metal cations under physiological conditions is crucial for designing effective metal chelators for biomedical applications, including detoxification, drug delivery, and metal overload disorders. Although formation constants (Table 1 and Table 2) provide an initial indication of ligand binding strength, relying solely on these values may lead to incomplete or misleading interpretations. The metal–ligand interaction is modulated by several competing equilibria and the medium composition (e.g., pH, ionic strength, presence of buffer ions), which can alter complex stability and speciation [35]. For this reason, a more comprehensive parameter, i.e., pL_0.5_, was used to evaluate the effective sequestering ability of each ligand. This parameter (pL_0.5_) represents the cologarithm of the ligand concentration required to sequester 50% of the trace metal ions present in solution and is described by the following Boltzmann-type equation [33]:

(6) $$\chi \;=\;\frac{1}{1+10^{\left(pL-pL0.5\right)}}$$

where χ is the sum of the mole fractions of the complex species and pL is the cologarithm of the total ligand analytical concentration.

**Figure 8:**
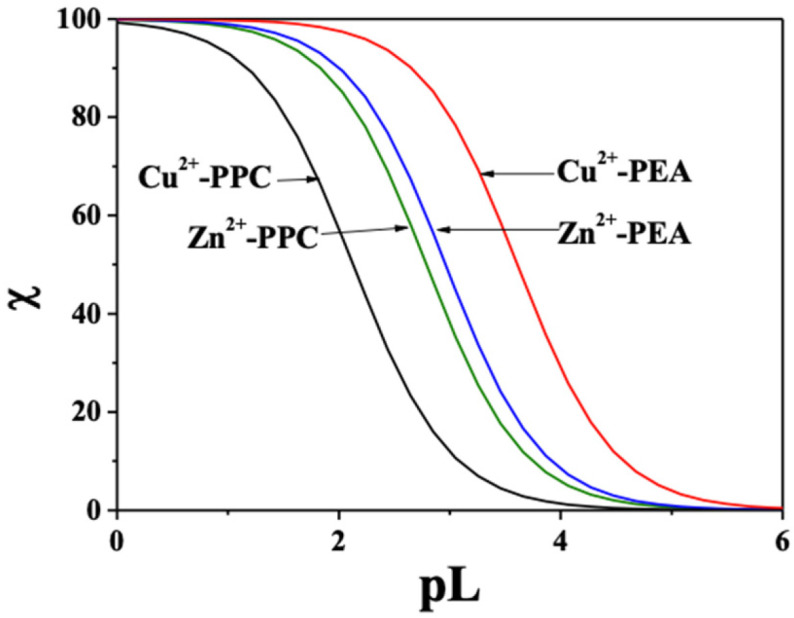
Comparison of the sequestering ability of PEA and PPC toward Cu^2+^ and Zn^2+^ at pH 7.4, t = 37 °C, and I = 0.15 mol L^−1^ in NaCl.

The pL_0_._5_ values, calculated at pH 7.4, I = 0.15 mol L^−1^ and different temperatures, are listed in Table S5 of Supplementary Materials. At 37 °C (physiological conditions), PEA shows significantly higher sequestering ability for both metal ions with pL_0_._5_ values of 3.61 for Cu^2+^ and 2.96 for Zn^2+^, compared to 2.13 and 2.79 for PPC, respectively. These findings are corroborated by the speciation diagrams (Figure 2 and Figure 3), which reveal that PEA forms a higher formation of complex species under physiological pH, reinforcing its higher complexing performance. Interestingly, PEA displays a greater affinity for Cu^2+^ than Zn^2+^, whereas PPC shows the opposite trend, demonstrating a higher sequestering capacity for Zn^2+^ and a lower one for Cu^2+^ (Figure 8). These findings support the strategic use of PEA as a more effective chelator in biomedical contexts, particularly in those systems requiring selective and efficient Cu^2+^ sequestration.

### 2.6. Quantum-Mechanical Calculations

With the aim of elucidating the sequestering ability carried by PEA and PPC toward Cu^2+^ and Zn^2+^, several molecular complexes were investigated by means of Density Functional Theory (DFT) calculations at the hybrid B3LYP/6-311++G(d,p) level supplemented by an agnostic analysis of the possible minima on the potential energy surfaces (PESs) of all complexes achieved by a thorough conformational analysis (see Section 3 for details). The latter exploration of the configurational space allowed us to avoid the possibility to calculate binding energies referred to molecular structures corresponding to local—and not to global—minima on the relatively complex PESs of the metal cation sequestering process, which have multiple possibilities to be hosted in the PEA and PPC molecular structures.

**Figure 9:**
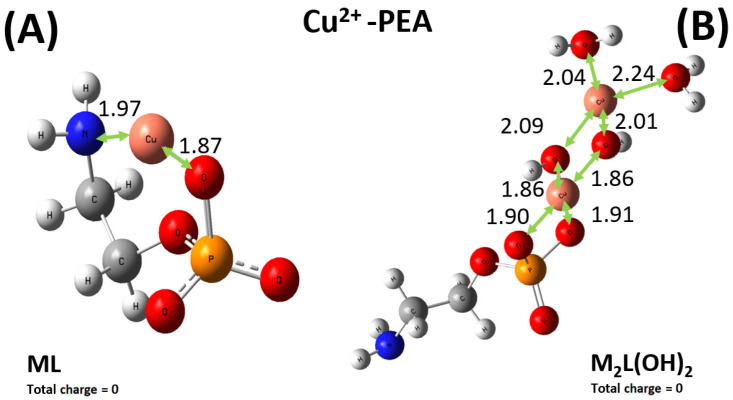
Global minimum structures of the ML^0^ (**A**) and M_2_L(OH)_2_^0^ (**B**) complexes formed by PEA and Cu^2+^, as predicted by conformational search followed by geometry optimization at the B3LYP/6-311++G(d,p) theory level. Red, silver, blue, white, orange, and pink color code refers to oxygen, carbon, nitrogen, hydrogen, phosphorus, and copper atoms. Relevant distances are reported in Å.

**Figure 10:**
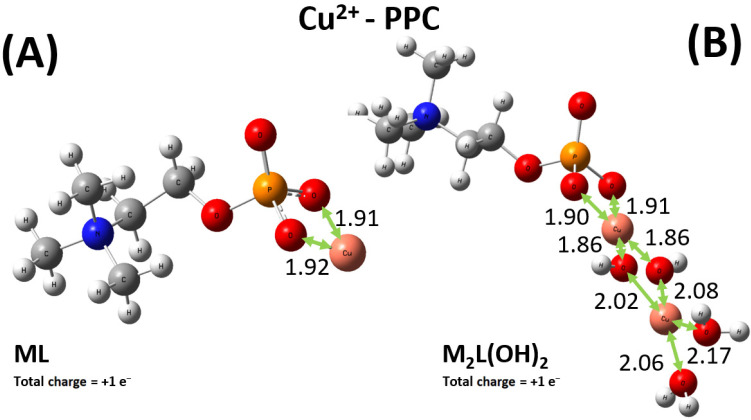
Global minimum structures of the ML^+^ (**A**) and M_2_L(OH)_2_^+^ (**B**) complexes formed by PPC and Cu^2+^, as predicted by conformational search followed by geometry optimization at the B3LYP/6-311++G(d,p) theory level. Red, silver, blue, white, orange, and pink color code refers to oxygen, carbon, nitrogen, hydrogen, phosphorus, and copper atoms. Relevant distances are reported in Å.

As shown in Figure 9A, Cu^2+^ preferably binds one oxygen atom of the phosphate group and the nitrogen atom of the amino group of the PEA structure. By contrast, the sequestering modalities preferred by the PPC molecules over the same cation involve two oxygen atoms of the phosphate group, as displayed in Figure 10A. Interestingly, although the relevant distances exhibited by the optimized structures may appear on average as comparable, the emerging binding energies of the ML complexes Cu^2+^-PEA and Cu^2+^-PPC are largely different. In fact, owing to the bridging action displayed by the Cu^2+^ cation in the PEA structure (Figure 9A), the binding energy featuring such a complex is about 60 kcal mol^−1^ stronger than its counterpart stabilizing the Cu^2+^-PPC complex. As also discussed in Section 2.1, our DFT-based simulations, combined with the exploration of the local configurational space, confirm that the Cu^2+^–PEA system forms more stable complexes than Cu^2+^–PPC under the ML coordination.

In addition, the experimental results reported above also indicate that under the M_2_L(OH)_2_, the complex formed by the PEA molecule with Cu^2+^ is more stable than the one established by PPC. Even though to a lesser extent with respect to the ML case, again the Cu^2+^-PEA moiety forms a Cu_2_L(OH)_2_^0^ complex which is about 7 kcal mol^−1^ more stable than the PPC counterpart, qualitatively in line with the experimental data. Furthermore, as also evidenced by the experiments where larger log*K* values were observed, it turns out that both M_2_L(OH)_2_ complexes formed by PEA and PPC with Cu^2+^ are measurably more favored (i.e., they exhibit larger binding energies) than the respective ML complexes, likely due to the direct involvement of water solvation and multiple bindings (see Figure 9B and Figure 10B). Incidentally, since in the di-nuclear Cu_2_L(OH)_2_ complexes both the ferromagnetic triplet and the antiferromagnetic (broken symmetry) singlet states are, in principle, possible, additional geometry optimizations of the respective triplet states have been executed. However, it turns out that the di-nuclear Cu_2_L(OH)_2_^0^ complex formed by PEA in the singlet state is by far the most stable structure, lying on a point of the potential energy surface more than 330 kcal mol^−1^ lower than its ferromagnetic counterpart. In addition, also in the PPC case, a very large energy difference of more than 390 kcal mol^−1^ has been recorded, with the singlet state representing the most stable electronic configuration. This way, only the singlet electronic states have been considered in the present analysis.

**Figure 11:**
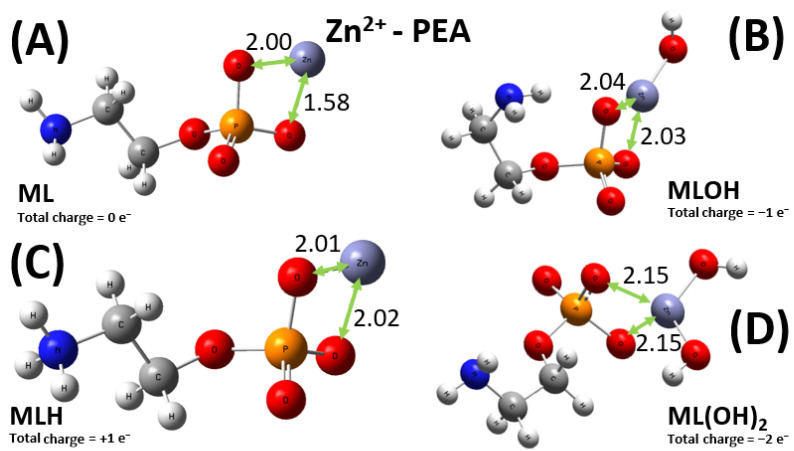
Global minimum structures of the ML^0^ (**A**), MLOH^−^ (**B**), MLH^+^ (**C**), and ML(OH)_2_^2−^ (**D**) complexes formed by PEA and Zn^2+^, as predicted by conformational search followed by geometry optimization at the B3LYP/6-311++G(d,p) theory level. Red, silver, blue, white, orange, and lilac color code refers to oxygen, carbon, nitrogen, hydrogen, phosphorus, and zinc atoms. Relevant distances are reported in Å.

**Figure 12:**
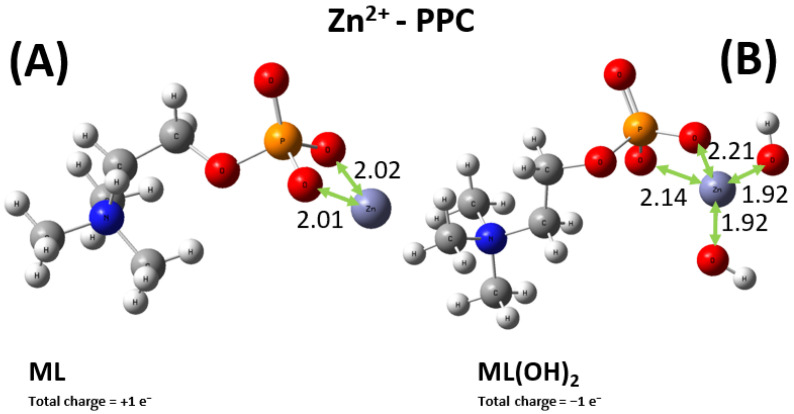
Global minimum structures of the ML^+^ (**A**) and ML(OH)_2_^−^ (**B**) complexes formed by PPC and Zn^2+^, as predicted by conformational search followed by geometry optimization at the B3LYP/6-311++G(d,p) theory level. Red, silver, blue, white, orange, and lilac color code refers to oxygen, carbon, nitrogen, hydrogen, phosphorus, and zinc atoms. Relevant distances are reported in Å.

As previously stated, the situation for the sequestering ability of PEA toward the Zn^2+^ cation is more intricate, with four relevant types of metal–ligand complexes, displayed in Figure 11. Among them, only the ML moiety allows for a direct comparison between the PEA and PPC case. In fact, as displayed in Figure 12 and as discussed in the experimental sections, additionally to the ML^+^ one, the only other relevant species for the Zn^2+^-PPC complex is the ML(OH)_2_^−^. It is worth noticing that whilst the Zn^2+^ cation is asymmetrically bound between two of the oxygen atoms of the phosphate group of the PEA ligand (Figure 11A), the same metal ion is evenly shared between two of these oxygen atoms in the PPC structure (Figure 12A). The different molecular modalities underlie a measurably different binding energy in the two cases. In fact, an extra binding energy of about 4 kcal mol^−1^ witnesses a slightly more efficient sequestering ability by the PEA molecule with respect to the PPC one toward the Zn^2+^ cation, a result consistent with the experimental evidence reported in the previous sections. In a nutshell, PEA is a better chelating agent toward the investigated bivalent metal cations than PPC.

Although the raw binding energy values of the remainder ligand–metal complexes (i.e., MLOH^−^, MLH^+^, and ML(OH)_2_^2−^ in the PEA case and the ML(OH)_2_^−^ only in the PPC one) are not that informative per se, the relative behavior across the whole data set of the binding energy values for all 10 complexes simulated here can deliver some information that, at least in principle, might be directly compared with, e.g., the log*K* experimental values. This kind of analysis would inform, inter alia, of the reliability of the conformational search analysis combined with the quantum-mechanical geometry optimizations here reported.

Affording a semi-quantitative correlation between quantum-mechanical data gathered from optimized geometries at 0 K (even though the conformational sampling was initially executed by approximating a thermal contribution of 25 °C) and under implicit solvation conditions with experimental ones obtained under realistic conditions is always challenging. In fact, solvation modalities of ligands, metal cations, and of the complexes they form typically involve complex and dynamical hydration shells which are completely neglected by any continuum solvation model. Coordinating water molecules are, by construction, not included in these models, and in our work only the di-nuclear Cu_2_L(OH)_2_ complexes (Figure 9B and Figure 10B) contain a partial explicit solvation. However, it is usually accepted that Cu^2+^ aqua complexes have a Cu(H_2_O)_n_^2+^ stoichiometry with n = 4–6 [36,37], as well as for Zn(H_2_O)_6_^2+^ aqua complex [38]. To include the different solvation shells, computationally demanding ab initio molecular dynamics (AIMD) simulations of the species investigated should be executed under periodic boundary conditions, as recently reported by our group for different systems [39,40,41]. In addition, since thermodynamic-related quantities have to be extracted, enhanced sampling techniques—such as metadynamics [42]—would have to be exploited, a circumstance further increasing the computational complexity of this task. Although this would certainly represent a very interesting topic for a future investigation, it also has to be considered that this procedure would require a massive lowering of the electronic structure description quality, typically at the Generalized Gradient Approximation (GGA) DFT level, which represents a suboptimal choice for the investigated systems.

**Figure 13:**
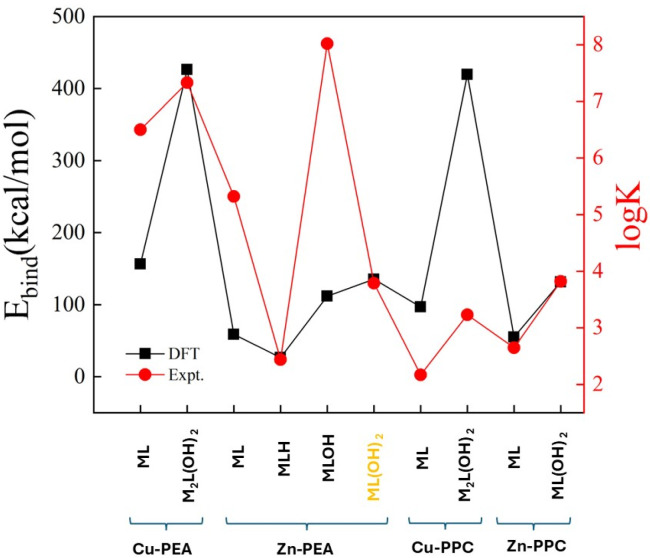
Correlations between the binding energy from DFT calculations (black squares) and the log*K* from experiments (red dots) over all the stable complexes formed by PEA and PPC ligands with the Cu^2+^ and Zn^2+^ cations. Notice that binding energies are reported with their absolute values whereas the complex marked in yellow (i.e., the Zn-PEA under the ML(OH)_2_^2−^ form) is the only case where an increase in the log*K* corresponds to a decrease in binding energy.

An attempt to establish a purely qualitative trend between computational and experimental data can be performed by considering the binding energies from our DFT-based simulations and the log*K* from experiments (Table 2). As reported in Figure 13, the relative trend of the data from our computations appears to be qualitatively correlated with the data emerging from the experiments carried out at 15 °C. In fact, each increase in the absolute value of the binding energy determined from DFT simulations corresponds to an increase in the log*K* experimental value, a circumstance reflecting an overall adherence of the respective results and corroborating the underlying atomistic mechanism of sequestration provided by the reported optimized molecular structures. Of course, this represents the best-case scenario and the qualitative correspondence between computational and experimental data has to be considered only within a given ligand–metal complex (i.e., its speciation structures) and not across the different complexes involving different ligands and metals. Moreover, the complex marked in yellow in Figure 13 (i.e., the Zn-PEA under the ML(OH)_2_^2−^ form) is the only case where an increase in the log*K* corresponds to a decrease in binding energy, signaling potential discrepancies in the overall data correlation for the respective structure.

### 2.7. Simulation Under Relevant Wilson’s Disease Conditions

Copper plays a critical role in Wilson’s disease (WD) [43], a genetic disorder characterized by impaired copper excretion, leading to toxic accumulation of free copper in plasma and cerebrospinal fluid (CSF), which contributes to neurological and hepatic damage [44]. To better understand copper speciation in these biological fluids under both physiological and pathological conditions, thermodynamic simulations were performed using experimentally determined stability constants for the Cu^2+^–PEA and Cu^2+^–PPC systems. For this purpose, two representative biological fluids, human plasma and cerebrospinal fluid (CSF), were selected and modeled based on realistic ionic compositions and physiologically relevant concentrations in WD. Simulations were carried out at pH 7.4, t = 37 °C, and I = 0.15 mol L^−1^, using literature-based values for the major ionic components [44,45,46]. For CSF, the composition included the following: [Na^+^] = 141 mmol L^−1^, [K^+^] = 2.9 mmol L^−1^, [Ca^2+^] = 1.25 μmol L^−1^, [Mg^2+^] = 1.2 μmol L^−1^, [Cl^−^] = 124 mmol L^−1^, [HCO_3_^−^] = 21 mmol L^−1^, [PO_4_^3−^] = 0.15 mmol L^−1^, [PEA] = 1 μmol L^−1^, [PPC] = 1 μmol L^−1^, and [Cu^2+^] = 1.6 μmol L^−1^. For plasma, the simulated composition was: [Na^+^] = 142 mmol L^−1^, [K^+^] = 4.6 mmol L^−1^, [Ca^2+^] = 2.5 mmol L^−1^, [Mg^2+^] = 0.9 mmol L^−1^, [Cl^−^] = 101 mmol L^−1^, [HCO_3_^−^] = 24 mmol L^−1^, [PO_4_^3−^] = 1.0 mmol L^−1^, [PEA] = 1.70 μmol L^−1^, [PPC] = 1.70 μmol L^−1^, and [Cu^2+^] = 3.2 μmol L^−1^. The simulations were performed using experimentally determined stability constants for Cu^2+^–PEA and Cu^2+^–PPC complexes obtained through potentiometric analysis, complemented by literature data on the interactions of PEA and PPC with key biologically relevant metal cations such as Mg^2+^ [4].

**Figure 14:**
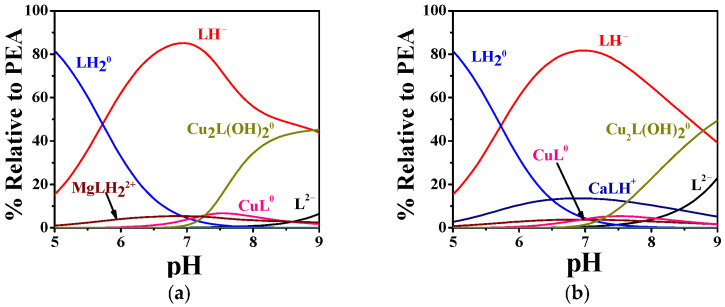
Speciation diagrams of PEA(L) complexes under (**a**) human plasma and (**b**) cerebrospinal fluid conditions in WD [44].

Under these conditions, PEA formed significant Cu^2+^ complexes in both fluids, while PPC showed no appreciable interaction. In plasma (Figure 14a), approximately 15% of the total copper was predicted to be bound, consisting of 5% ML^0^ (CuL^0^) and 10% M_2_L(OH)_2_^0^ complexes (Cu_2_L(OH)_2_^0^). In CSF (Figure 14b), the total Cu^2+^–PEA complexation increased to ~20%, with 5% ML^0^ and 15% M_2_L(OH)_2_^0^, indicating a slightly more favorable environment for Cu^2+^ binding in CSF. In contrast, simulations for the Cu^2+^–PPC system revealed no significant species under either plasma or CSF conditions, due to lower affinity of PPC for Cu^2+^.

**Figure 15:**
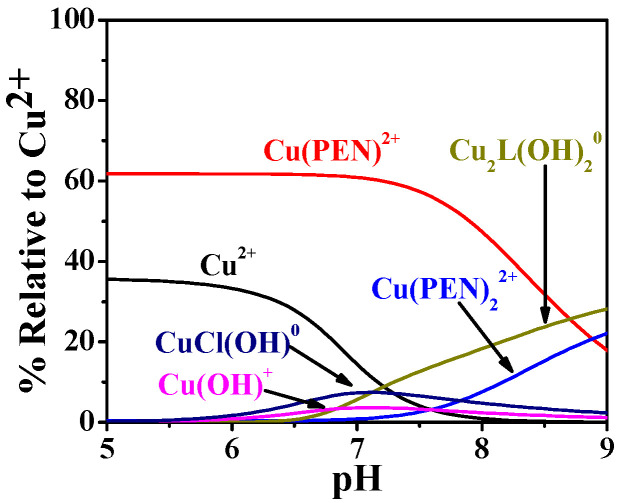
Speciation diagrams of PEA(L) and penicillamine (PEN) complexes under human plasma conditions in WD.

To further assess the Cu^2+^-binding capacity of PEA, an additional plasma simulation was performed in the presence of penicillamine (PEN), the first-line chelation therapy for WD. Based on literature data, two Cu^2+^–PEN complexes (CuPEN^2+^ and Cu(PEN)_2_^2+^) were considered, with formation constants of logβ = 9.5 and 16.9 at 35 °C and I = 0.1 mol L^−1^ [47]; unfortunately, no values are available at 37 °C. Assuming a PEN plasma concentration of 28 µg L^−1^ after an 800 mg oral dose (therapeutic oral dose for WD) [48], the simulation indicates that, under physiological pH, 62% of Cu^2+^ is complexed with PEN (forming CuPEN^2+^), while 17% remains bound to PEA. Notably, formation of the Cu(PEN)_2_^2+^ complex involved only 2% of Cu^2+^. This comparative analysis demonstrates that, although PEN exhibits greater efficiency in Cu^2+^ complexation, PEA retains a significant Cu^2+^-binding capacity, indicating that it still contributes under physiological plasma conditions. The data further suggest that PEA can participate in Cu^2+^ sequestration even in the presence of classical chelators. To allow direct comparison of Cu^2+^ complexation between the two ligands (PEA and PEN), the results shown in Figure 15 are expressed as formation percentages relative to the total Cu^2+^ species.

## 3. Materials and Methods

### 3.1. Materials

Solutions of the ligands, O-phosphorylethanolamine (PEA) and O-phosphorylcholine chloride (PCC), were obtained by weighing and dissolving the corresponding products (Sigma-Aldrich/Merck, Darmstadt, Germany, ≥99%). Solutions of the metals were prepared by weighing and dissolving the respective salts, cupric chloride dihydrate (Fluka/Chemie GmbH, Buchs, Switzerland, puriss. p.a. ACS; ≥99%), and zinc chloride (Sigma-Aldrich/Chemie GmbH, Steinheim, Germany, puriss. p.a. ACS reagent; ≥98%). Both metal solutions were standardized by titration with EDTA (ethylenediaminetetraacetic acid disodium salt dihydrate, Sigma-Aldrich, BioUltra, 99%) standard solution. Sodium chloride (NaCl) solutions were prepared by weighing the salt (Sigma-Aldrich/Merck, Darmstadt, Germany, ≥99%), previously dried at 110 °C. Solutions of hydrochloric acid (HCl) and sodium hydroxide (NaOH) were obtained by diluting the Fluka vials (Fluka/Honeywell, Charlotte, North Carolina, United States) and standardized by titration with sodium carbonate and potassium acid phtalate, respectively. Both these last salts were purchased by Sigma-Aldrich/Merck, Darmstadt, Germany, ≥99.5% and pre-dried at t = 110 °C before their use.

### 3.2. Potentiometric Equipment and Procedure

Potentiometric measurements were performed as titrations using Metrohm–Titrando 809 automated potentiometer, equipped with a combined glass electrode ORION (type Ross 8102SC) and a Metrohm Dosino 800 automatic dispenser. The titrations were carried out in thermostated cells with a capacity of 25 mL and connected to D1-G Haake thermostat. The titration system is interfaced with a PC by Metrohm TIAMO 2.0 software, which acquires experimental data (mL/mV), tracking the e.m.f. stability and monitoring other parameters, such as titrant delivery and data acquisition. Estimated accuracy of this system is ±0.15 mV and ±0.002 mL for e.m.f. and reading of titrant volume. The potentiometric titrations consist of adding standard NaOH to 25 mL of solutions containing metal cations, Cu^2+^ or Zn^2+^ (1 ≤ C_M_/mmol L^−1^ ≤ 2), PEA or PCC (1 ≤ C_L_/mmol L^−1^ ≤ 4) at different concentration ratios (0.33 ≤ C_M_/C_L_ ≤ 1), HCl (2 ≤ C_H_^+^/mmol L^−1^ ≤ 8), and NaCl as a supporting electrolyte. The potentiometric titrations were performed under different conditions of temperature (15 ≤ t/°C ≤ 37 at I = 0.15 mol L^−1^) and ionic strength (0.15 ≤ I/mol L^−1^ ≤ 0.97 at t = 25 °C) while constant magnetic stirring and nitrogen bubbling occurring for homogeneity of the solutions without -O_2_ and -CO_2_ interference. The experimental conditions of the potentiometric titrations were given in Table S6 in Supplementary Materials. For each measurement, under the same experimental temperature and ionic strength conditions, independent titrations of HCl with standard NaOH were performed to determine the standard electrode potential (E^0^) and pK_w_ values.

### 3.3. ^1^H NMR Equipment and Procedure

^1^H NMR measurements were recorded as titrations by a Varian NMR spectrometer 500 MHz using 1,4-dioxane (0.1%) as internal reference (δ_CH dioxane_ = 3.70 ppm), referring all chemical shifts (δ) to tetramethylsilane (TMS) and the coupling constants, J, in Hz. To reduce water signal, presaturation technique was adopted in a 9:1 H_2_O:D_2_O solution at t = 25 °C. The titrations were first performed in 25 mL of NaCl solution (I = 0.15 mol L^−1^) containing the ligands (PEA, 5.5 mmol L^−1^ or PPC, 6.4 mmol L^−1^), varying the pH by adding NaOH. Then, titrations carried out in 25 mL of NaCl solution (I = 0.15 mol L^−1^) containing the ligands (PEA or PPC in the same above concentrations) and Zn^2+^ (5.6 and 3.6 mmol L^−1^), respectively. Again, the pH of the solutions was increased by addition of NaOH. The experimental conditions adopted for the Cu^2+^-PEA/PPC and Zn^2+^-PEA/PPC systems are given in Table S6 (Supplementary Materials).

### 3.4. Post-Processing Calculations

Experimental potentiometric data were processed by BSTAC and STACO programs [49], with which the formation constant values and other parameters (analytical concentrations of the reagents, standard potential E^0^, junction potential) were determined. The dependence of the formation constant values on the ionic strength and temperature were studied by LIANA software [49]. ^1^H NMR data were processed by HypNMR2008, through which the formation constant values were calculated along with the individual chemical shift values of the species by analyzing the observed signals and fast mutual exchange in the NMR time scale [50]. The speciation diagrams and formation percentage of the species were given by the Hyss program [51].

### 3.5. Quantum-Mechanical Calculations

PEA and PPC molecules not only offer multiple binding sites to divalent metal cations such as Cu^2+^ and Zn^2+^, but also, they are sufficiently complex, in terms of degrees of freedom, to exhibit multifaceted potential energy surfaces showing several local minima. As a seminal step of our investigation, the CREST software version 3.0.1 (Conformer–Rotamer Ensemble Sampling Tool) [52] was employed to identify the lowest-energy conformer through semi-empirical methods such as GFN*n*-xTB [53]. Such an approach was adopted on the complexes binding Cu^2+^ and Zn^2+^. Once the three most stable configurations for each investigated complex were selected, a finer analysis was executed to identify the PES global minimum using the Gaussian 16 software [54] which, based on Density Functional Theory (DFT), proved to be effective in defining the ground-state geometries of PEA and PPC, along with several of their derivatives for the complexation with Cu^2+^ and Zn^2+^ ions.

In this work, all DFT calculations were performed using the B3LYP [55,56,57] hybrid exchange and correlation functional, with 20% of exact exchange. Especially for *d*-block metals, the specific choice of the exchange and correlation functional in the modeling of coordination complexes is delicate due to the neglect of static correlation effects. In fact, there exists a relatively vast literature on the performances of different DFT functionals for systems such as bis(μ-oxo) and bis(μ-hydroxo) di-metal—such as the M_2_L(OH)_2_ complexes investigated in the current work. The interested reader can refer to Refs. [58,59,60,61]. Geometry optimization of the molecular structures emerging from the conformational search analysis was performed by employing the 6-311++G(d,p) atomic basis sets for all atoms. As far as the simulation of the solvent is concerned, the CPCM (Conductive Polarizable Continuum Model) model [62] was employed by setting parameters mimicking the water electrostatics. After structural relaxation to the ground state, vibrational calculations were performed not only to establish the correctness of the previous calculations (i.e., absence of imaginary frequencies), but also to obtain the zero-point energy (ZPE) associated with each optimized molecular structure. Nuclear quantum effects have to be taken into account carefully in proton transfer phenomena because of their relevance in water also at ambient conditions [63,64].

## 4. Conclusions

The comparative analysis of Cu^2+^ and Zn^2+^ complexation with the two biologically relevant ligands, PEA and PPC, reveals marked differences in their coordination behavior and stability profiles. For Cu^2+^ containing systems, potentiometric data showed that both ligands form ML and M_2_L(OH)_2_ complexes, but the stability of those containing PEA was significantly greater. The stability constants are higher for both complexes in the Cu^2+^–PEA system under all tested conditions, suggesting a stronger metal–ligand interaction. Similarly, Zn^2+^ complexation showed that PEA forms a more complex array of complexes, including MLH^+^ and MLOH^−^, in addition to ML^0^ and ML(OH)_2_^2−^, while PPC only forms ML^+^ and ML(OH)_2_^−^. The log*K* values consistently showed higher stability for PEA complexes, confirming a superior affinity.

The ^1^H NMR titration data corroborate these findings, demonstrating significant chemical shift variations in the Zn^2+^–PEA system, indicative of diverse coordination equilibria. In contrast, the Zn^2+^–PPC system showed minimal chemical shift variation, supporting the presence of fewer complexes. Importantly, the experimental data and speciation models derived from ^1^H NMR were consistent with those obtained from potentiometric titrations, reinforcing the robustness of the speciation analysis.

Thermodynamic analyses using van’t Hoff and Debye-Hückel models provided additional insight. Cu^2+^–PEA complexation is entropically favored, aligning with electrostatic interaction mechanisms. Zn^2+^–PEA binding, particularly in hydrolyzed forms, involves more exothermic enthalpy changes.

The sequestering ability, evaluated via pL_0.5_, highlighted that PEA outperforms PPC in effectively binding both metal cations under physiological conditions. This trend is most pronounced for Cu^2+^, for which PEA displays a much higher pL_0.5_ value, suggesting potential utility in chelation therapies for copper-related pathologies. Interestingly, PPC exhibited slightly better performance than PEA in Zn^2+^ sequestration under specific conditions, which may indicate selective ligand applications depending on the metal of interest.

DFT calculations combined with conformational analysis reveal that PEA is a more effective chelator than PPC for both Cu^2+^ and Zn^2+^. Cu^2+^ binds more strongly to PEA due to its bridging coordination between the amino and phosphate groups, resulting in a binding energy ~60 kcal mol^−1^ higher than in the PPC complex. Similar trends are observed in the M_2_L(OH)_2_ forms, with PEA complexes remaining more stable by ~7 kcal mol^−1^. For Zn^2+^, the ML^0^ complex with PEA is ~4 kcal mol^−1^ more stable than its PPC counterpart, owing to asymmetric coordination. While one Zn^2+^–PEA complex (ML(OH)_2_^2−^) shows a deviation from experimental data, the overall trend of the computationally derived binding energies aligns well with the experimentally measured log*K* values. These results confirm the stronger metal-binding affinity of PEA and support its enhanced sequestering capability, in agreement with experimental observations.

Simulation in real fluid conditions have been conducted to evaluate the ability of PEA and PPC to form appreciable complex species under relevant physiological conditions. Results show a total complexation with Cu^2+^ reaching 15% in plasma and 20% in CSF. This finding gains relevance in the context of WD, a genetic disorder characterized by impaired hepatic copper excretion leading to systemic copper overload. In WD patients, copper accumulates not only in the liver but also leaks into the bloodstream and eventually crosses into the CSF, where it contributes to neurotoxicity. Importantly, much of this copper exists in a non-ceruloplasmin-bound form, often referred to as free Cu^2+^, which is more chemically reactive and biologically harmful. The simulations suggest that, under elevated copper conditions such as those observed in WD, endogenous ligands like PEA, present in both plasma and CSF, could contribute to buffering this labile copper fraction. The formation of stable Cu^2+^–PEA complexes, especially under CSF conditions, points to a potential physiological role of PEA in modulating free copper levels and limiting its redox activity. Conversely, the absence of any significant complexation with PPC further emphasizes the selectivity and functional relevance of PEA in copper coordination. These results support the hypothesis that metal-binding metabolites such as PEA might act as part of the brain’s intrinsic detoxification or regulatory mechanisms, particularly in pathologies involving disrupted copper homeostasis like WD.

## Figures and Tables

**Table 1:** Formation constant values of Cu^2+^-PEA and Cu^2+^-PPC **complexes** at different temperature and ionic strength conditions, obtained by potentiometry.

				logβ ^a^		
Complex	L	t = 15 °C		t = 25 °C		t = 37 °C
		I = 0.15 ^b^	I = 0.15 ^b^	I = 0.49 ^b^	I = 0.97 ^b^	I = 0.15 ^b^
ML^0^	PEA	6.50(6) ^c^	5.63(7) ^c^	6.01(4) ^c^	6.58(7) ^c^	6.31(9) ^c^
M_2_L(OH)_2_^0^		−3.80(9)	−4.55(6)	−3.92(3)	−2.96(5)	−3.35(10)
						
ML^+^	PPC	2.17(5)	2.05(3)	1.57(9)	2.16(9)	2.39(9)
M_2_L(OH)_2_^+^		−7.90(2)	−6.83(2)	−6.14(4)	−5.60(2)	−7.18(9)
				log*K* ^d^		
ML^0^	PEA	6.50	5.63	6.01	6.58	6.31
M_2_L(OH)_2_^0^		7.33	6.17	6.83	7.81	6.99
						
ML^+^	PPC	2.17	2.05	1.57	2.16	2.39
M_2_L(OH)_2_^+^		3.23	3.89	4.65	5.17	3.16

^a^ Reaction (1); ^b^ mol L^−1^; ^c^ ≥95% interval of confidence; ^d^ reactions (2) and (3).

**Table 2:** Formation constant values of Zn^2+^-PEA and Zn^2+^-PPC complexes at various temperature and ionic strength conditions obtained by potentiometry.

				logβ ^a^		
Complex	L	t = 15 °C		t = 25 °C		t = 37 °C
		I = 0.15 ^b^	I = 0.15 ^b^	I = 0.48 ^b^	I = 0.97 ^b^	I = 0.15 ^b^
MLH^+^	PEA	12.82(8) ^c^	12.32(5) ^c^	12.24(4) ^c^	12.39(7) ^c^	12.68(5) ^c^
ML^0^		5.32(5)	4.29(5)	4.46(8)	4.10(9)	4.90(5)
MLOH^−^		−2.48(3)	−2.37(2)	−2.79(2)	−2.91(3)	−2.09(4)
ML(OH)_2_^2−^		−13.47(9)	−12.55(3)	−13.10(3)	−13.67(7)	−12.17(5)
						
ML^+^	PPC	2.65(8)	2.41(4)	2.01(6)	1.62(9)	2.51(4)
ML(OH)_2_^−^		−13.28(6)	−12.68(2)	−12.94(2)	−13.42(3)	−12.23(2)
				log*K* ^d^		
MLH^+^	PEA	2.44	2.18	2.17	2.30	2.84
ML^0^		5.32	4.29	4.46	4.10	4.90
MLOH^−^		8.02	6.78	6.28	5.97	6.29
ML(OH)_2_^2−^		3.79	4.55	3.96	3.25	4.93
						
ML^+^	PPC	2.65	2.41	2.01	1.62	2.51
ML(OH)_2_^−^		3.82	4.42	4.12	3.50	4.87

^a^ Reaction (1); ^b^ mol L^−1^; ^c^ ≥95% interval of confidence; ^d^ reactions (2) and (3).

**Table 3:** Comparison of formation constant values of Zn2+-PEA and Zn2+-PPC complexes obtained by potentiometric and 1H-NMR titrations, at t = 25 °C and I = 0.15 mol L−1 in NaCl.

L	Complex	logβ ^a^	
		^1^H-NMR	Potentiometry
PEA	MLH^+^	12.1(2) ^b^	12.32
	ML^0^	4.29	4.29
	MLOH^−^	−2.37	−2.37
	ML(OH)_2_^2−^	−12.55	−12.55
			
PPC	ML^+^	2.4(4)	2.41
	ML(OH)_2_^−^	−12.68	−12.68

^a^ Reaction (1); ^b^ ≥95% interval of confidence.

**Table 4:** ΔG, ΔH, TΔS of M-L complexes at t = 25 °C and I = 0.15 mol L^−1^ in NaCl, along with formation constants at infinite dilution and parameter C for the dependence on the ionic strength at t = 25 °C.

M	L	Complex ^a^	ΔG ^b^	ΔH ^b^	TΔS ^b^	z*	logβ^0 a^	C
Cu^2+^	PEA	ML^0^	−32.1	58(8) ^c^	90	8	6.38(11) ^c^	1.92(16) ^c^
		M_2_L(OH)_2_^0^	26.0	29(13)	3	10	−3.69(12)	2.89(18)
								
Cu^2+^	PPC	ML^+^	−11.7	14(8)	26	4	2.81(19)	0.9(4)
		M_2_L(OH)_2_^+^	38.8	59(8)	20	6	−5.84(17)	2.4(4)
								
Zn^2+^	PEA	MLH^+^	−70.3	−4(5)	66	8	13.24(8)	0.72(12)
		ML^0^	−24.5	−27(12)	−3	8	5.36(12)	0.49(25)
		ML(OH)^−^	13.5	32(9)	9	6	−1.67(4)	−0.04(7)
		ML(OH)_2_^2−^	71.6	95(13)	24	2	−12.15(8)	−1.18(9)
								
Zn^2+^	PPC	ML^+^	−13.8	−13(12)	1	4	3.45(2)	−0.19(4)
		ML(OH)_2_^−1^	72.4	82(9)	10	2	−12.31(2)	−0.68(3)

^a^ Reaction (1); ^b^ kJ mol^−1^; ^c^ ≥95% interval of confidence.

## Data Availability

The original contributions presented in the study are included in the article/Supplementary Material, further inquiries can be directed to the corresponding authors.

## Supplementary Materials

The following supporting information can be downloaded at: https://www.mdpi.com/article/10.3390/molecules30193923/s1, Table S1: Protonation constant values of PEA and PPC at various temperature and ionic strength conditions; Table S2: Hydrolysis constants of Cu^2+^ and formation constants of Cu^2+^-Cl^−^ complex at different temperatures and ionic strength values; Table S3: Hydrolysis constants of Zn^2+^ at different temperatures and ionic strength values; Table S4: Chemical shift (ppm) Zn^2+^-PEA e Zn^2+^-PPC complex nuclei at t = 25 °C and I = 0.15 mol L^−1^ in NaCl; Table S5: pL_0.5_ values at various temperature conditions (pH = 7.4, I = 0.15 mol L^−1^ in NaCl); Table S6: Experimental conditions of potentiometric and ^1^H-NMR titrations of M(Cu^2+^, Zn^2+^)-L(PEA, PPC) systems; Table S7: Formation constant values of all the complexes considered for simulations under real physiological conditions (t = 37 °C and I = 0.15 mol L^−1^); Figure S1: Comparison between experimental (observed) and calculated chemical shift values for (A) Zn^2+^–PEA and (B) Zn^2+^–PPC complexes [4,65,66,67,68].

## Abbreviations

The following abbreviations are used in this manuscript:

CPCM	Conductive Polarizable Continuum Model
CREST	Conformer–Rotamer Ensemble Sampling Tool
CSF	Cerebrospinal fluid
DFT	Density Functional Theory
PEA	O-phosphorylethanolamine
PESs	Potential Energy Surfaces
PPC	O-phosphorylcholine
TMS	Tetramethylsilane
ZPE	Zero-Point Energy
NDs	Neurodegenerative Diseases
AD	Alzheimer’s Disease
WD	Wilson’s Disease
MD	Menkes Disease
PEN	Penicillamine

## References

[1] Takeda H., Takahashi M., Hara T., Izumi Y., Bamba T. (2019). Improved quantitation of lipid classes using supercritical fluid chromatography with a charged aerosol detector. J. Lipid Res..

[2] Cardoso R.M.S., Lairion F., Disalvo E.A., Loura L.M.S., Moreno M.J. (2024). Dipole Potential of Monolayers with Biologically Relevant Lipid Compositions. Molecules.

[3] Holdaway C.M., Leonard K.A., Nelson R., van der Veen J., Das C., Watts R., Clugston R.D., Lehner R., Jacobs R.L. (2025). Alterations in phosphatidylethanolamine metabolism impacts hepatocellular lipid storage, energy homeostasis, and proliferation. Biochim. Biophys. Acta Mol. Cell Biol. Lipids.

[4] Aiello D., Cordaro M., Napoli A., Foti C., Giuffrè O. (2022). Speciation Study on O-Phosphorylethanolamine and O-Phosphorylcholine: Acid-Base Behavior and Mg^2+^ Interaction. Front. Chem..

[5] Nuschy L., Sarkar B., Zamyatina A., Wilson I.B.H. (2025). Substrate flexibility of *Mycoplasma fermentans* mf1 phosphorylcholine transferase. Glycoconj. J..

[6] Labrada K.P., Strobl S., Eckmair B., Blaukopf M., Dutkiewicz Z., Hykollari A., Malzl D., Paschinger K., Yan S., Wilson I.B.H. (2020). Zwitterionic Phosphodiester-Substituted Neoglycoconjugates as Ligands for Antibodies and Acute Phase Proteins. ACS Chem. Biol..

[7] Zhang Y., Jen F.E.C., Fox K.L., Edwards J.L., Jennings M.P. (2023). The biosynthesis and role of phosphorylcholine in pathogenic and nonpathogenic bacteria. Trends Microbiol..

[8] Chung Y.-C., Chen I.H., Chen C.-J. (2008). The surface modification of silver nanoparticles by phosphoryl disulfides for improved biocompatibility and intracellular uptake. Biomaterials.

[9] Hui S.C.N., Zöllner H.J., Oeltzschner G., Edden R.A.E., Saleh M.G. (2022). In vivo spectral editing of phosphorylethanolamine. Magn. Reson. Med..

[10] Cuellar J., Parada-Díaz L., Garza J., Mejía S.M. (2023). A Theoretical Analysis of Interaction Energies and Intermolecular Interactions between Amphotericin B and Potential Bioconjugates Used in the Modification of Nanocarriers for Drug Delivery. Molecules.

[11] Zheng D., Lu Z.G., Li J., Dong J., Zhang X., Zhang X., Cao D. (2025). Unveiling the Interaction Mechanism of siRNA with Lipid Bilayers of Different Types for siRNA-Based Therapy. J. Phys. Chem. B.

[12] Karabaliev M., Paarvanova B., Savova G., Tacheva B., Jahn S., Georgieva R. (2024). Electrochemical Investigation of the Stability of Poly-Phosphocholinated Liposomes. Molecules.

[13] Yoshizaki Y., Konno T. (2023). Cellular Internalization and Exiting Behavior of Zwitterionic 4-Armed Star-Shaped Polymers. Molecules.

[14] Wang Y., Wilfahrt D., Jonker P., Lontos K., Cai C., Cameron B., Xie B., Peralta R., Schoedel E., Gunn W. (2025). Tumour interstitial fluid-enriched phosphoethanolamine suppresses T cell function. Nat. Cell Biol..

[15] Abate C., Aiello D., Cordaro M., Giuffrè O., Napoli A., Foti C. (2022). Binding ability of l-carnosine towards Cu^2+^, Mn^2+^ and Zn^2+^ in aqueous solution. J. Mol. Liq..

[16] Tyczyńska M., Gędek M., Brachet A., Stręk W., Flieger J., Teresiński G., Baj J. (2024). Trace Elements in Alzheimer’s Disease and Dementia: The Current State of Knowledge. J. Clin. Med..

[17] Chen L., Shen Q., Liu Y., Zhang Y., Sun L., Ma X., Song N., Xie J. (2025). Homeostasis and metabolism of iron and other metal ions in neurodegenerative diseases. Signal Transduct. Target. Ther..

[18] Gromadzka G., Tarnacka B., Flaga A., Adamczyk A. (2020). Copper Dyshomeostasis in Neurodegenerative Diseases—Therapeutic Implications. Int. J. Mol. Sci..

[19] Zhang H.-L., Wang X.-C., Liu R. (2022). Zinc in Regulating Protein Kinases and Phosphatases in Neurodegenerative Diseases. Biomolecules.

[20] Zhang Y., Gao H., Zheng W., Xu H. (2022). Current understanding of the interactions between metal ions and Apolipoprotein E in Alzheimer’s disease. Neurobiol. Dis..

[21] Esmieu C., Hostachy S., Hureau C. (2025). Cu(I) chelators: Useful tools to reveal and control Cu(I) homeostasis and toxicity. Coord. Chem. Rev..

[22] Di Natale G., Sabatino G., Sciacca M.F.M., Tosto R., Milardi D., Pappalardo G. (2022). Aβ and Tau Interact with Metal Ions, Lipid Membranes and Peptide-Based Amyloid Inhibitors: Are These Common Features Relevant in Alzheimer’s Disease?. Molecules.

[23] Banik S.P., Bagchi D., Banerjee P., Chakraborty S., Bagchi M., Bose C., De D., Saha S., Chakraborty S. (2025). Subtle concentration changes in zinc hold the key to fibrillation of α-synuclein: An updated insight on the micronutrient’s role in prevention of neurodegenerative disorders. Front. Mol. Biosci..

[24] Rulmont C., Stigliani J.-L., Hureau C., Esmieu C. (2024). Rationally Designed Cu(I) Ligand to Prevent CuAβ-Generated ROS Production in the Alzheimer’s Disease Context. Inorg. Chem..

[25] Okafor M., Gonzalez P., Ronot P., El Masoudi I., Boos A., Ory S., Chasserot-Golaz S., Gasman S., Raibaut L., Hureau C. (2022). Development of Cu(ii)-specific peptide shuttles capable of preventing Cu–amyloid beta toxicity and importing bioavailable Cu into cells. Chem. Sci..

[26] Fijałkowski P., Pomastowski P., van Eldik R., Rafińska K. (2025). Multifunctional role of Lactoferrin in metal ion interactions and biomedical applications: A review. Int. J. Biol. Macromol..

[27] Shen X., Sheng H., Zhang Y., Dong X., Kou L., Yao Q., Zhao X. (2024). Nanomedicine-based disulfiram and metal ion co-delivery strategies for cancer treatment. Int. J. Pharm. X.

[28] Maiti B.K., Moura J.J.G. (2021). Diverse biological roles of the tetrathiomolybdate anion. Coord. Chem. Rev..

[29] Šegota S., Vojta D., Pletikapić G., Baranović G. (2015). Ionic strength and composition govern the elasticity of biological membranes. A study of model DMPC bilayers by force- and transmission IR spectroscopy. Chem. Phys. Lipids.

[30] Indelicato S., Bongiorno D., Calabrese V., Perricone U., Almerico A.M., Ceraulo L., Piazzese D., Tutone M. (2017). Micelles, Rods, Liposomes, and Other Supramolecular Surfactant Aggregates: Computational Approaches. Interdiscip. Sci. Comput. Life Sci..

[31] Filella M., May P.M. (2005). Reflections on the calculation and publication of potentiometrically-determined formation constants. Talanta.

[32] Carnamucio F., Foti C., Micale N., Van Pelt N., Matheeussen A., Caljon G., Giuffrè O. (2024). Metronidazole Interaction with Cu^2+^ and Zn^2+^: Speciation Study in Aqueous Solution and Biological Activity Evaluation. ACS Omega.

[33] Foti C., Giuffrè O. (2020). Interaction of Ampicillin and Amoxicillin with Mn^2+^: A Speciation Study in Aqueous Solution. Molecules.

[34] Mazur T., Malik M., Bieńko D.C. (2024). The impact of chelating compounds on Cu^2+^, Fe^2+^/^3+^, and Zn^2+^ ions in Alzheimer’s disease treatment. J. Inorg. Biochem..

[35] De Stefano C., Gianguzza A., Pettignano A., Piazzese D., Sammartano S. (2011). Uranium(VI) sequestration by polyacrylic and fulvic acids in aqueous solution. J. Radioanal. Nucl. Chem..

[36] de Almeida K.J., Rinkevicius Z., Hugosson H.W., Ferreira A.C., Ågren H. (2007). Modeling of EPR parameters of copper(II) aqua complexes. Chem. Phys..

[37] Pavelka M., Burda J.V. (2005). Theoretical description of copper Cu(I)/Cu(II) complexes in mixed ammine-aqua environment. DFT and ab initio quantum chemical study. Chem. Phys..

[38] Nazmutdinov R.R., Schmickler W., Kuznetsov A.M. (2005). Microscopic modelling of the reduction of a Zn(II) aqua-complex on metal electrodes. Chem. Phys..

[39] Carnamucio F., Foti C., Cordaro M., Saija F., Cassone G., da Rocha S.R.P., Giuffrè O. (2024). Metal Complexation for the Rational Design of Gemcitabine Formulations in Cancer Therapy. ACS Appl. Mater. Interfaces.

[40] Abate C., Giuffrè O., Amadeo A., Saija F., Cassone G., Foti C. (2024). Experimental and computational study on morin and its complexes with Mg^2+^, Mn^2+^, Zn^2+^, and Al^3+^: Coordination and antioxidant properties. J. Inorg. Biochem..

[41] Giacobello F., Mollica-Nardo V., Foti C., Ponterio R.C., Saija F., Trusso S., Sponer J., Cassone G., Giuffrè O. (2022). Hydrolysis of Al^3+^ in Aqueous Solutions: Experiments and Ab Initio Simulations. Liquids.

[42] Bussi G., Laio A. (2020). Using metadynamics to explore complex free-energy landscapes. Nat. Rev. Phys..

[43] Feng H., Fu Q., Du W., Zhu R., Ge X., Wang C., Li Q., Su L., Yang H., Song J. (2021). Quantitative Assessment of Copper(II) in Wilson’s Disease Based on Photoacoustic Imaging and Ratiometric Surface-Enhanced Raman Scattering. ACS Nano.

[44] Gromadzka G., Grycan M., Przybyłkowski A.M. (2023). Monitoring of Copper in Wilson Disease. Diagnostics.

[45] Artru A., Cottrell J.E., Young W.L. (2010). Cerebrospinal fluid. Cottrell’s Neuroanesthesia.

[46] Barrett H.B.K., Boitano S., Barman S. (2013). Ganongs Review of Medical Physiology.

[47] Gupta V.K., Ali I. (1998). Determination of stability constants of Fe(II), Co(II) and Cu(II)–nitrilotriacetate–penicillamine mixed complexes by electrophoresis. Talanta.

[48] Aronson J.K. (2006). Meyler’s Side Effects of Drugs: The International Encyclopedia of Adverse Drug Reactions and Interactions.

[49] De Stefano C., Sammartano S., Mineo P., Rigano C., Gianguzza A., Pelizzetti E., Sammartano S. (1997). Marine Chemistry—An Environmental Analytical Chemistry Approach.

[50] Frassineti C., Alderighi L., Gans P., Sabatini A., Vacca A., Ghelli S. (2003). Determination of protonation constants of some fluorinated polyamines by means of ^13^C NMR data processed by the new computer program HypNMR2000. Protonation sequence in polyamines. Anal. Bioanal. Chem..

[51] Alderighi L., Gans P., Ienco A., Peters D., Sabatini A., Vacca A. (1999). Hyperquad simulation and speciation (HySS): A utility program for the investigation of equilibria involving soluble and partially soluble species. Coord. Chem. Rev..

[52] Pracht P., Grimme S., Bannwarth C., Bohle F., Ehlert S., Feldmann G., Gorges J., Müller M., Neudecker T., Plett C. (2024). CREST—A program for the exploration of low-energy molecular chemical space. J. Chem. Phys..

[53] Bannwarth C., Ehlert S., Grimme S. (2019). GFN2-xTB—An Accurate and Broadly Parametrized Self-Consistent Tight-Binding Quantum Chemical Method with Multipole Electrostatics and Density-Dependent Dispersion Contributions. J. Chem. Theory Comput..

[54] Frisch M.J., Trucks G.W., Schlegel H.B., Scuseria G.E., Robb M.A., Cheeseman J.R., Scalmani G., Barone V., Petersson G.A., Nakatsuji H. (2016). Gaussian 16, Revision C.01.

[55] Vosko S.H., Wilk L., Nusair M. (1980). Accurate spin-dependent electron liquid correlation energies for local spin density calculations: A critical analysis. Can. J. Phys..

[56] Lee C., Yang W., Parr R.G. (1988). Development of the Colle-Salvetti correlation-energy formula into a functional of the electron density. Phys. Rev. B Condens. Matter.

[57] Stephens P.J., Devlin F.J., Chabalowski C.F., Frisch M.J. (1994). Ab Initio Calculation of Vibrational Absorption and Circular Dichroism Spectra Using Density Functional Force Fields. J. Phys. Chem..

[58] Cramer C.J., Truhlar D.G. (2009). Density functional theory for transition metals and transition metal chemistry. Phys. Chem. Chem. Phys..

[59] Perrella F., Langellotti V., Buttarazzi E., Cucciolito M.E., Melchiorre M., Pinto G., Prokopenko V., Rega N., Ruffo F., Petrone A. (2024). Unveiling Stereo-Electronic Effects in Homogeneous Catalysis Integrating Theory and Experiments: The Potential of Dimeric Iron(III) Salen Complexes in Methyl Levulinate Transesterification. ChemCatChem.

[60] Bühl M., Kabrede H. (2006). Geometries of Transition-Metal Complexes from Density-Functional Theory. J. Chem. Theory Comput..

[61] David G., Wennmohs F., Neese F., Ferré N. (2018). Chemical Tuning of Magnetic Exchange Couplings Using Broken-Symmetry Density Functional Theory. Inorg. Chem..

[62] Tomasi J., Mennucci B., Cammi R. (2005). Quantum Mechanical Continuum Solvation Models. Chem. Rev..

[63] Cassone G. (2020). Nuclear Quantum Effects Largely Influence Molecular Dissociation and Proton Transfer in Liquid Water under an Electric Field. J. Phys. Chem. Lett..

[64] Dasgupta S., Cassone G., Paesani F. (2025). Nuclear Quantum Effects and the Grotthuss Mechanism Dictate the pH of Liquid Water. J. Phys. Chem. Lett..

[65] Giuffrè O., Aiello D., Chillè D., Napoli A., Foti C. (2020). Binding ability of arsenate towards Cu^2+^ and Zn^2+^: Thermodynamic behavior and simulation under natural water conditions. Environ. Sci. Process. Impacts.

[66] Crea F., Falcone G., Foti C., Giuffrè O., Materazzi S. (2014). Thermodynamic data for Pb^2+^ and Zn^2+^ sequestration by biologically important S-donor ligands, at different temperatures and ionic strengths. New J. Chem..

[67] Crea F., De Stefano C., Milea D., Pettignano A., Sammartano S. (2015). SALMO and S3M: A Saliva Model and a Single Saliva Salt Model for Equilibrium Studies. Bioinorg. Chem. Appl..

[68] Aiello D., Carnamucio F., Cordaro M., Foti C., Napoli A., Giuffrè O. (2021). Ca^2+^ Complexation With Relevant Bioligands in Aqueous Solution: A Speciation Study With Implications for Biological Fluids. Front. Chem..

