# Supplementary material for: Comprehensive Speciation and Computational Study of Cu2+ and Zn2+ Complexation with O-Phosphorylethanolamine and O-Phosphorylcholine in Aqueous Solution
Source: Molecules. 2025 Sep 29;30(19):3923. doi: 10.3390/molecules30193923 (PMC12525833; doi:10.3390/molecules30193923)
Supplement: Supplementary file 1 [file molecules-30-03923-s001.zip › molecules-3816512-supplementary.pdf]

Supplementary Material

Table S1. Protonation constant values of PEA and PPC at various temperature and ionic strength conditions.

L	Reaction	t /°C	I / mol L^{-1}	logβ[1]
PEA	$L + H^+ = LH^+$	15	0.15	10.381
		25	0.15	10.141
		25	0.5	10.071
		25	1	10.087
		37	0.15	9.836
	$L + 2H^+ = LH_2^{2+}$	15	0.15	16.021
		25	0.15	15.731
		25	0.5	15.607
		25	1	15.551
		37	0.15	15.560
	$L + 3H^+ = LH_3^{3+}$	15	0.15	17.08
		25	0.15	16.69
		25	0.5	16.79
		25	1	16.45
		37	0.15	17.29
PPC	$L^+ + H^+ = LH^{2+}$	15	0.15	5.635
		25	0.15	5.646
		25	0.5	5.542
		25	1	5.459
		37	0.15	5.668
	$L^2 + 2H^+ = LH_2^{3+}$	15	0.15	-
		25	0.15	6.53
		25	0.5	6.23
		25	1	6.12
		37	0.15	6.71

[1]D. Aiello, M. Cordaro, A. Napoli, C. Foti and O. Giuffrè, Speciation Study on O-Phosphorylethanolamine and O-Phosphorylcholine: Acid–Base Behavior and Mg^{2+} Interaction. Front. Chem. 2022, 10, 864648.

Table S2. Hydrolysis constants of Cu^{2+} and formation constants of Cu^{2+}-Cl^- complexes at different temperatures and ionic strength values.

Reaction	t /°C	I / mol L^{-1}	logβ[1]
$Cu^{2+} + H_2O = Cu(OH)^+ + H^+$	15	0.15	-7.9
	25	0.15	-7.7
	25	0.5	-7.7
	25	1	-7.7
	37	0.15	-7.5
$2Cu^{2+} + H_2O = Cu_2(OH)^{3+} + H^+$	15	0.15	-6.41
	25	0.15	-6.10
	25	0.5	-6.10
	25	1	-6.79
	37	0.15	-5.88
$2Cu^{2+} + 2H_2O = Cu_2(OH)_2^{2+} + 2H^+$	15	0.15	-11.13
	25	0.15	-10.72
	25	0.5	-10.72
	25	1	-10.65
	37	0.15	-10.36
$2Cu^{2+} + 4H_2O = Cu_2(OH)_4^0 + 4H^+$	15	0.15	-22.43
	25	0.15	-21.36
	25	0.5	-21.36
	25	1	-21.36
	37	0.15	-20.7
$Cu^{2+} + Cl^- = CuCl^+$	15	0.15	-0.26
	25	0.15	-0.24
	25	0.5	-0.23
	25	1	-0.15
	37	0.15	-8.54
$Cu^{2+} + 2Cl^- = CuCl_2^0$	15	0.15	-0.93
	25	0.15	-0.9
	25	0.5	-1.07
	25	1	-1.0
	37	0.15	-0.19
$Cu^{2+} + 3Cl^- = CuCl_3^-$	15	0.15	-1.95
	25	0.15	-1.94
	25	0.5	-2.27
	25	1	-2.19
	37	0.15	-0.76
$Cu^{2+} + Cl^- + H_2O = CuClOH^0 + H^+$	15	0.15	-6.55
	25	0.15	-6.28
	25	0.5	-6.24
	25	1	-6.34
	37	0.15	-5.98

[1] O. Giuffrè, D. Aiello, D. Chillè, A. Napoli, C. Foti, Binding ability of arsenate towards Cu^{2+} and Zn^{2+}: thermodynamic behavior and simulation under natural water conditions, *Environ. Sci.: Processes Impacts*, 2020, 22, 1731.

Table S3. Hydrolysis constants of Zn^{2+} at different temperatures and ionic strength values.

Reaction	t /°C	I / mol L^{-1}	logβ[1]
$Zn^{2+} + H_2O = Zn(OH)^{+} + H^{+}$	15	0.15	-9.5
	25	0.15	-9.14
	25	0.5	-9.15
	25	1	-9.16
	37	0.15	-8.78
$Zn^{2+} + 2H_2O = Zn(OH)_2^{0} + 2H^{+}$	15	0.15	-17.64
	25	0.15	-17.10
	25	0.5	-17.1
	25	1	-17.22
	37	0.15	-16.52
$Zn^{2+} + 3H_2O = Zn(OH)_3^{-} + 3H^{+}$	15	0.15	-29.12
	25	0.15	-28.4
	25	0.5	-28.4
	25	1	-28.47
	37	0.15	-27.54
$Zn^{2+} + 4H_2O = Zn(OH)_4^{2-} + 4H^{+}$	15	0.15	-41.67
	25	0.15	-40.40
	25	0.5	-40.85
	25	1	-40.38
	37	0.15	-39.47
$2Zn^{2+} + H_2O = Zn_2(OH)^{3+} + H^{+}$	15	0.15	-9.27
	25	0.15	-8.70
	25	0.5	-8.89
	25	1	-8.89
	37	0.15	-8.54
$2Zn^{2+} + 6\ H_2O = Zn_2(OH)_6^{2-} + 6H^{+}$	15	0.15	-58.91
	25	0.15	-57.50
	25	0.5	-57.53
	25	1	-57.32
	37	0.15	-55.9

[1] F. Crea, G. Falcone, C. Foti, O. Giuffrè, S. Materazzi. Thermodynamic data for Pb^{2+} and Zn^{2+} sequestration by biologically important S-donor ligands, at different temperatures and ionic strengths, New J. Chem., 2014, 38, 3973.

Table S4. Chemical shift (ppm) Zn^{2+}-PEA and Zn^{2+}-PPC complex nuclei at t = 25°C and I = 0.15 mol L^{-1} in NaCl.

L		MLH	ML	MLOH	ML(OH)2
**PEA**	$\delta_{CH_{2(1)}}$	3.9(8) [1]	3.7(8) [1]	4.6(8) [1]	–
	$\delta_{CH2(2)}$	3.15(2)	2.71(2)	3.58(2)	–
**PPC**	$\delta_{CH_{2(1)}}$	–	4.12(2) [a)]	–	4.11(2) [1]
	$\delta_{CH2(2)}$	–	3.54(4)	–	3.53(4)

[1] ≥95% Interval of Confidence.

Table S5. $pL_{0.5}$ values at various temperature conditions (pH = 7.4, I = 0.15 mol L^{-1} in NaCl).

		$pL_{0.5}$		
**M**	**L**	**t = 15°C**	**t = 25°C**	**t = 37°C**
**Cu^{2+}**	**PEA**	3.39	2.67	3.61
	**PPC**	2.04	1.83	2.13
**Zn^{2+}**	**PEA**	2.47	2.35	2.96
	**PPC**	2.67	2.57	2.79

Table S6. Experimental conditions of potentiometric and ^{1}H-NMR titrations of **M** (Cu^{2+}, Zn^{2+}) – **L** (PEA, PPC) systems.

**Potentiometry**					
**t /°C**	**I / mol L^{-1}**		**C_M /mmol L^{-1}**	**C_L /mmol L^{-1}**	**M:L**
15, 25, 37	0.15		1-2	1-4	0.33-1
25	0.48		1-2	1-4	0.33-1
25	0.97		1-2	1-4	0.33-1
**^{1}H NMR (t = 25°C and I = 0.15 mol L^{-1})**					
**Zn^{2+}-PEA**			**Zn^{2+}-PPC**		
**C_M /mmol L^{-1}**	**C_L /mmol L^{-1}**	**M:L**	**C_M /mmol L^{-1}**	**C_L /mmol L^{-1}**	**M:L**
5.6	5.5	1.02	3.6	6.4	0.6

Table S7. Formation constant values of all the species considered for simulations under real physiological conditions (t = 37°C and I = 0.15 mol L^{-1}).

Reaction	logβ[1]	Reaction	logβ[1]
$H^+ + OH^- = H_2O$	-13.77	$Na^+ + K^+ + PO_4^{3-} + H^+ = NaKHPO_4^0$	12.45
$Mg^{2+} + H_2O = Mg(OH)^+ + H^+$	-11.45	$Mn^{2+} + H_2O = MnOH^- + H^+$	-9.20
$Ca^{2+} + H_2O = Ca(OH)^+ + H^+$	-12.86	$2Mn^{2+} + 3H_2O = Mn_2(OH)_3^- + 3H^+$	-23.51
$PEA^{2-} + H^+ = PEAH^-$	9.836[3]	$Cu^{2+} + H_2O = CuOH^+ + H^+$	-7.5
$PEA^{2-} + 2\ H^+ = PEAH^0$	15.56[3]	$2Cu^{2+} + H_2O = Cu_2(OH)^{3+} + H^+$	-5.88
$PEA^{2-} + 3\ H^+ = PEAH^+$	17.29[3]	$2Cu^{2+} + 2H_2O = Cu_2(OH)_2^{2+} + 2H^+$	-10.36
$Ca^{2+} + PEA^{2-} = CaPEA^0$	1.57[4]	$2Cu^{2+} + 4H_2O = Cu_2(OH)_4^0 + 4H^+$	-20.7
$Ca^{2+} + PEA^{2-} + H^+ = CaPEAH^+$	11.27[4]	$Cu^{2+} + Cl^- = CuCl^+$	-8.54
$Mg^{2+} + PEA^{2-} = MgPEA^0$	1.94[3]	$Cu^{2+} + 2Cl^- = CuCl_2^0$	-0.19
$Mg^{2+} + PEA^{2-} + H^+ = MgPEAH^+$	11.65[3]	$Cu^{2+} + 3Cl^- = CuCl_3^-$	-0.76
$Mg^{2+} + PEA^{2-} + 2H^+ = MgPEAH_2^{2+}$	16.96[3]	$Cu^{2+} + Cl^- + H_2O = CuClOH^0 + H^+$	-5.98
$Cu^{2+} + PEA^{2-} = CuPEA^0$	6.31[2]	$Zn^{2+} + H_2O = ZnOH^+ + H^+$	-10.02
$Cu^{2+} + PEA^{2-} + OH^- = Cu(PEA)OH^-$	-3.35[2]	$Zn^{2+} + 2H_2O = Zn(OH)_2^0 + 2H^+$	-16.52
$PO_4^{3-} + H^+ = HPO_4^{2-}$	11.64	$Zn^{2+} + 3H_2O = Zn(OH)_3^- + 3H^+$	-27.54
$PO_4^{3-} + 2H^+ = H_2PO_4^-$	18.47	$Zn^{2+} + 4H_2O = Zn(OH)_4^{2-} + 4H^+$	-39.47
$PO_4^{3-} + 3H^+ = H_3PO_4^0$	20.50	$2Zn^{2+} + H_2O = Zn_2(OH)^{3+} + H^+$	-8.54
$Mg^{2+} + PO_4^{3-} + H^+ = MgHPO_4^0$	13.72	$2Zn^{2+} + 6\ H_2O = Zn_2(OH)_6^{2-} + 6H^+$	-55.9
$Mg^{2+} + PO_4^{3-} + 2H^+ = MgH_2PO_4^+$	19.67	$Mg^{2+} + CO_3^{2-} = MgCO_3^0$	2.22
$Ca^{2+} + PO_4^{3-} + H^+ = CaHPO_4^0$	13.58	$Mg^{2+} + CO_3^{2-} + H^+ = MgHCO_3^+$	10.56
$Ca^{2+} + PO_4^{3-} + 2H^+ = CaH_2PO_4^+$	19.54	$Ca^{2+} + CO_3^{2-} = CaCO_3^0$	2.56
$Na^+ + PO_4^{3-} = NaPO_4^{2-}$	0.95	$Ca^{2+} + CO_3^{2-} + H^+ = CaHCO_3^+$	10.86
$Na^+ + PO_4^{3-} + H^+ = NaHPO_4^-$	12.41	$Na^+ + CO_3^{2-} = NaCO_3^-$	0.80
$Na^+ + PO_4^{3-} + 2H^+ = NaH_2PO_4^0$	18.69	$Na^+ + CO_3^{2-} + H^+ = NaHCO_3^0$	9.87
$2Na^+ + PO_4^{3-} = Na_2PO_4^-$	1.75	$K^+ + CO_3^{2-} = KCO_3^-$	0.61
$2Na^+ + PO_4^{3-} + H^+ = Na_2HPO_4^0$	12.13	$K^+ + CO_3^{2-} + H^+ = KHCO_3^0$	9.79
$K^+ + PO_4^{3-} = KPO_4^{2-}$	0.85	$Mg^{2+} + Cl^- = MgCl^+$	0.18
$K^+ + PO_4^{3-} + H^+ = KHPO_4^-$	12.22	$Ca^{2+} + Cl^- = CaCl^+$	0.03
$K^+ + PO_4^{3-} + 2H^+ = KH_2PO_4^0$	18.49	$Na^+ + Cl^- = NaCl^0$	-0.5
$2K^+ + PO_4^{3-} = K_2PO_4^-$	1.39	$K^+ + Cl^- = KCl^0$	-0.48
$2K^+ + PO_4^{3-} + H^+ = K_2HPO_4^0$	12.16	$CO_3^{2-} + H^+ = HCO_3^-$	9.85
$Na^+ + K^+ + PO_4^{3-} = NaKPO_4^-$	1.93	$CO_3^{2-} + 2H^+ = H_2CO_3^0$	15.97

[1] Crea, F.; De Stefano, C., Milea, D., Pettignano, A., Sammartano, S, SALMO and S3M: A Saliva Model and a Single Saliva Salt Model for Equilibrium Studies, Bioinorg. Chem. Appl. 2015.
[2] This work.
[3] Aiello, D.; Cordaro, M.; Napoli, A.; Foti, C.; Giuffrè, O.; Speciation Study on O-Phosphorylethanolamine and O-Phosphorylcholine: Acid–Base Behavior and Mg^{2+} Interaction. Front. Chem. 2022, 10:864648.
[4] Aiello, D.; Carnamucio, F.; Cordaro, M.; Foti, C.; Napoli, A.; Giuffrè, O. Ca^{2+} Complexation With Relevant Bioligands in Aqueous Solution: A Speciation Study With Implications for Biological Fluids. Front Chem. 2021, 24;9:640219.

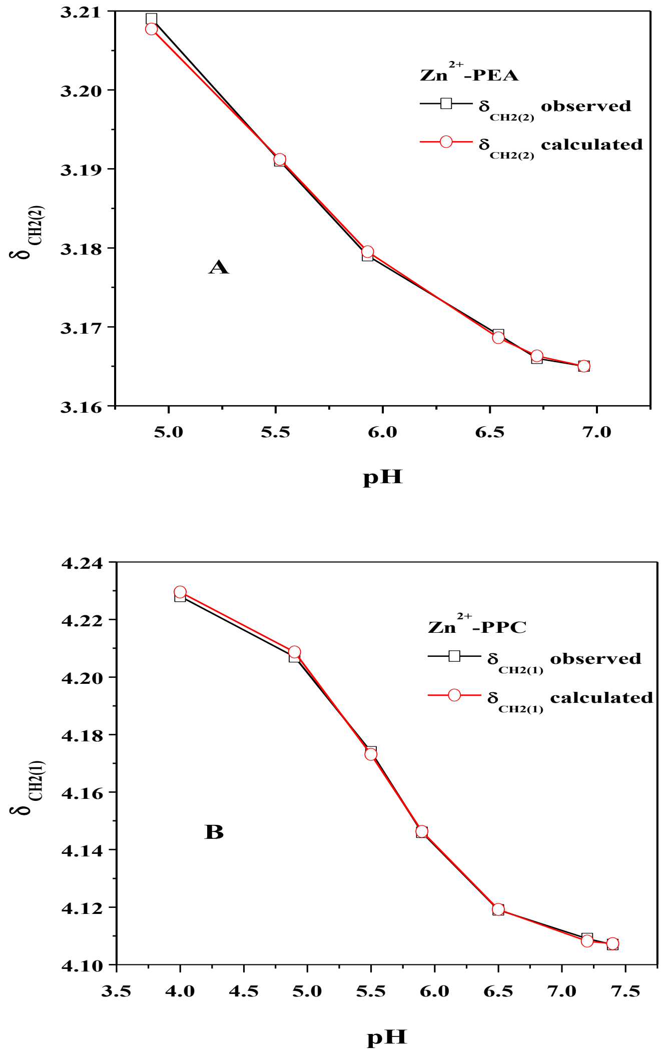

Figure S1A-B. Comparison between experimental (observed) and calculated chemical shift values for: **(A)** Zn^{2+}–PEA and **(B)** Zn^{2+}–PPC complexes.
